# Nanobody cocktails potently neutralize SARS-CoV-2 D614G N501Y variant and protect mice

**DOI:** 10.1073/pnas.2101918118

**Published:** 2021-04-23

**Authors:** Phillip Pymm, Amy Adair, Li-Jin Chan, James P. Cooney, Francesca L. Mordant, Cody C. Allison, Ester Lopez, Ebene R. Haycroft, Matthew T. O’Neill, Li Lynn Tan, Melanie H. Dietrich, Damien Drew, Marcel Doerflinger, Michael A. Dengler, Nichollas E. Scott, Adam K. Wheatley, Nicholas A. Gherardin, Hariprasad Venugopal, Deborah Cromer, Miles P. Davenport, Raelene Pickering, Dale I. Godfrey, Damian F. J. Purcell, Stephen J. Kent, Amy W. Chung, Kanta Subbarao, Marc Pellegrini, Alisa Glukhova, Wai-Hong Tham

**Affiliations:** ^a^Infectious Diseases and Immune Defences Division, The Walter and Eliza Hall Institute of Medical Research, Parkville, VIC 3052, Australia;; ^b^Department of Medical Biology, University of Melbourne, Melbourne, VIC 3010, Australia;; ^c^Department of Microbiology and Immunology, The Peter Doherty Institute for Infection and Immunity, University of Melbourne, Melbourne, VIC 3000, Australia;; ^d^Australian Research Council Centre for Excellence in Convergent Bio-Nano Science and Technology, University of Melbourne, Melbourne, VIC 3010, Australia;; ^e^Australian Research Council Centre of Excellence in Advanced Molecular Imaging, University of Melbourne, Melbourne, VIC 3010, Australia;; ^f^Department of Biochemistry and Molecular Biology, Monash University, Clayton, VIC 3800, Australia;; ^g^Kirby Institute, University of New South Wales, Sydney, NSW 2052, Australia;; ^h^Department of Mathematics and Statistics, University of New South Wales, Sydney, NSW 2052, Australia;; ^i^Department of Diabetes, Central Clinical School, Monash University, Melbourne, VIC 3004, Australia;; ^j^WHO Collaborating Centre for Reference and Research on Influenza, The Peter Doherty Institute for Infection and Immunity, University of Melbourne, Melbourne, VIC 3000, Australia;; ^k^Drug Discovery Biology, Monash Faculty of Pharmacy and Pharmaceutical Sciences, Monash University, Parkville, VIC 3052, Australia;; ^l^Department of Biochemistry and Pharmacology, University of Melbourne, Melbourne, VIC 3010, Australia

**Keywords:** SARS-CoV-2, nanobodies, crystallography, cryo-EM, antiviral therapeutics

## Abstract

Neutralizing antibodies are important for immunity against SARS-CoV-2 and as therapeutics for the prevention and treatment of COVID-19. Here, we identified high-affinity nanobodies from alpacas immunized with coronavirus spike and receptor-binding domains (RBD) that disrupted RBD engagement with the human receptor angiotensin-converting enzyme 2 (ACE2) and potently neutralized SARS-CoV-2. Epitope mapping, X-ray crystallography, and cryo-electron microscopy revealed two distinct antigenic sites and showed two neutralizing nanobodies from different epitope classes bound simultaneously to the spike trimer. Nanobody-Fc fusions of the four most potent nanobodies blocked ACE2 engagement with RBD variants present in human populations and potently neutralized both wild-type SARS-CoV-2 and the N501Y D614G variant at concentrations as low as 0.1 nM. Prophylactic administration of either single nanobody-Fc or as mixtures reduced viral loads by up to 10^4^-fold in mice infected with the N501Y D614G SARS-CoV-2 virus. These results suggest a role for nanobody-Fc fusions as prophylactic agents against SARS-CoV-2.

## Significance

Neutralizing antibodies are important for immunity against severe acute respiratory syndrome coronavirus 2 (SARS-CoV-2) and as therapeutics for the prevention and treatment of COVID-19. We identified high-affinity nanobodies against SARS-CoV-2 receptor-binding domain and found that nanobody cocktails consisting of two noncompeting nanobodies were able to block ACE2 engagement with RBD variants present in human populations and potently neutralize both wild-type SARS-CoV-2 and the N501Y D614G variant at low concentrations. Prophylactic administration of nanobody cocktails reduced viral loads in mice infected with the N501Y D614G SARS-CoV-2 virus, showing that nanobody cocktails are useful as prophylactic agents against SARS-CoV-2.

Coronaviruses are enveloped RNA viruses that infect many mammalian and avian species. The current devastating COVID-19 pandemic is caused by severe acute respiratory syndrome coronavirus 2 (SARS-CoV-2), which has resulted in over 90 million infections and over 2 million deaths worldwide. The severe acute respiratory syndrome coronavirus (SARS-CoV) and the Middle East respiratory syndrome coronavirus (MERS-CoV) are also highly pathogenic human pathogens. SARS-CoV resulted in the SARS epidemic in 2002 with over 8,000 infections and a 10% fatality rate, and MERS-CoV resulted in the MERS epidemic in 2012 with over 877 infections and a ∼36% fatality rate.

Entry into a host cell is the critical first step in the viral life cycle. Of the four major structural proteins encoded by the coronavirus genome, the spike protein plays a crucial role in viral attachment, fusion, and entry (1, 2). The spike protein of SARS-CoV-2 is present on the virion surface as a trimer and mediates recognition of human angiotensin-converting enzyme 2 (ACE2) on the surface of host cells, subsequently triggering membrane fusion (3–6). The receptor-binding domain (RBD) localized on the N-terminal subunit (S1) mediates receptor binding and is a target of neutralizing antibodies (7–14). The SARS-CoV-2 RBD spans residues 319 to 541 with the receptor-binding motif (RBM) spanning residues 438 to 506, which contains most of the contacting residues of SARS-CoV-2 that bind to human ACE2 (6, 15). Recently, three SARS-CoV-2 variants of concern, B.1.1.7, B.1.351, and B.1.1.248, are known to carry several mutations within the spike protein. In particular, all three variants share one specific mutation called D614G, which replaced the initial SARS-CoV-2 strain to become the dominant form of the virus circulating globally and is thought to promote transmission of the virus but with no impact on pathogenesis (16). Within the RBD, B.1.1.7 carries N501Y mutation; B.1.351 carries K417N, E484K, and N501Y mutations; and B.1.1.248 carries K417N/T, E484K, and N501Y mutations (17, 18). E484K has recently been demonstrated to reduce virus neutralization for several monoclonal antibodies and polyclonal human sera (19–21). N501 is one of six key contact residues within the RBD, and the N501Y mutation increases binding affinity to human and mouse ACE2 (22, 23). The spike proteins from SARS-CoV-2 and SARS-CoV are ∼80% identical, and both RBDs bind ACE2 (6, 15, 24). Antiviral therapies against human coronaviruses that block receptor engagement and the ability to undergo membrane fusion will effectively inhibit the process of viral entry and block infection (25).

Alpacas, llamas, and camels have evolved one of the smallest naturally occurring antigen recognition domains called nanobodies (26). Nanobodies are ∼12 to 15 kDa in size, highly stable across a wide range of pH and temperature, display strong binding affinities to target proteins, and can be expressed with high yields in bacterial, yeast, and mammalian expression systems (27, 28). In particular, due to their stability, nanobodies are highly suited for development as potential bio-inhaled therapies against respiratory diseases (29). Indeed, Ablynx developed an inhaled anti–respiratory syncytial virus nanobody, ALX-0171, which has robust antiviral effects and reduces symptoms of virus infection in animal models (30). Recently, neutralizing nanobodies against SARS-CoV-2 were identified using several approaches, including immunization of llamas, yeast surface display of synthetic nanobodies, and phage display of naïve llama nanobody library or humanized synthetic nanobody library (31–40). These nanobodies were readily engineered into different multivalent forms, fused to Fc domains, and affinity matured to increase neutralization potency (32, 33, 36, 37, 40). These studies also show that nanobodies and their derivatives retain function either upon lyophilization, heat treatment, or aerosolization, suggesting a potential avenue for development of inhaled therapeutics against COVID-19 (32, 33).

While most nanobodies show potent neutralization against SARS-CoV-2 using in vitro assays, very few studies have examined the in vivo efficacy of nanobodies for the prevention and treatment for COVID-19 (41, 42). Due to their small size, nanobodies are rapidly cleared through renal elimination, which poses challenges for in vivo studies (43). However, the short half-life of nanobodies can be overcome via fusion to larger proteins such as albumin or to the Fc fragment of IgG (41–43). Furthermore, to the best of our knowledge, no study has examined the potential for nanobody mixtures in the prevention of COVID-19 in vivo, which may be advantageous for controlling more highly infectious variants and reducing the potential for virus escape mutations to develop (7). Due to their unique properties, it will be important to further understand the potential of nanobodies as therapeutics against SARS-CoV-2.

By screening nanobody phage display libraries generated from two alpacas immunized with coronavirus spike and RBDs, we identified a collection of nanobodies that bound to RBD with low nanomolar (nM) affinities, inhibited RBD-ACE2 complex formation, and neutralized SARS-CoV-2. X-ray crystallography, cryogenic electron microscopy (cryo-EM), and epitope binning experiments identified several combinations of noncompeting nanobodies as potential antibody mixture combinations. Nanobody-Fc fusions effectively blocked ACE2 receptor engagement with naturally occurring RBD variants present in human populations, showed potent neutralization against wild-type (WT) SARS-CoV-2 and an N501Y D614G variant, and, when used prophylactically, protected mice infected with a SARS-CoV-2 N501Y D614G variant.

## Results

### High-Affinity RBD-Binding Nanobodies that Block ACE2 Engagement and Neutralize Virus.

**Fig. 1.**
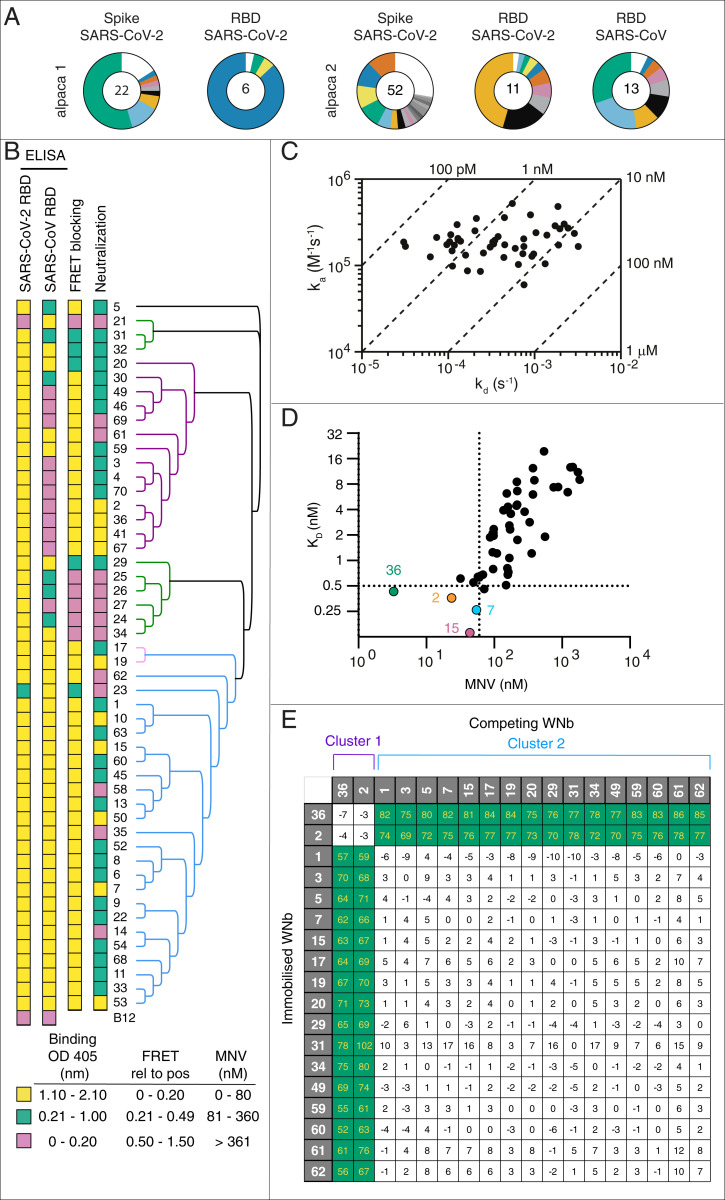
Identification and functional characterization of neutralizing anti-RBD nanobodies. (*A*) The distribution of nanobody sequences from five phage panning campaigns against spike and RBD proteins. The number in the inner circle indicates the number of distinct clonal groups based on the CDR3 sequences. White indicates sequences isolated only once, and colored pie slices are proportional to the number of clonally related sequences. (*B*) Heatmap of nanobody ELISA binding to SARS-CoV-2 and SARS-CoV RBD, blocking activity by FRET-based assay and neutralization potency with the MNV assay. Nanobodies are ordered based on their clonal lineage as shown on the right. (*C*) Iso-affinity plot showing the dissociation rate constants (*K*_*D*_) and association rate constants (*K*_*a*_) of WNb nanobodies as measured by bio-layer interferometry. Symbols that fall on the same diagonal lines have the same equilibrium dissociation rate constants (*K*_D_) indicated on the top and right sides of the plot. (*D*) Comparison of potencies in the MNV assay with nanobody affinities with the four WNb lead candidates highlighted, which have antibody affinities <0.5 nM and neutralization activity <60 nM. (*E*) Epitope competition experiments using bio-layer interferometry using immobilized nanobodies indicated on the left column, incubated with nanobodies indicated on the top row, preincubated with SARS-CoV-2 RBD using a 10:1 molar ratio. Binding of RBD premixed with nanobody was calculated relative to RBD binding alone, which was assigned to 100%. The green and white boxes represent noncompeting and competing nanobodies, respectively.

To identify nanobodies that are effective at neutralizing SARS-CoV-2, we immunized two alpacas with combinations of recombinant spike and RBD proteins using a 42 d immunization schedule (*SI Appendix*, Fig. S1). Postimmunization sera showed higher reactivity to the respective antigens relative to preimmunization sera (*SI Appendix*, Fig. S1). The first alpaca was immunized with SARS-CoV-2 spike and RBD, and the second alpaca was immunized with SARS-CoV-2 spike, SARS-CoV-2 RBD, and SARS-CoV RBD (Fig. 1*A*). We generated nanobody phage display libraries from the two alpacas and performed two rounds of phage display on each antigen. Sequence alignment and phylogenetic analyses of nanobodies from the five campaigns, which were further verified by enzyme-linked immunosorbent assay (ELISA)-based screening for nanobodies that bound specifically to RBD, sequencing, and expression, led to the identification of 50 unique nanobodies (Fig. 1 *A* and *B* and *SI Appendix*, Table S1). We expressed and purified the 50 nanobodies and confirmed by ELISA that 48 bound to SARS-CoV-2 RBD (Fig. 1*B* and *SI Appendix*, Table S2). Furthermore, 31 bound SARS-CoV RBD at a similar level of reactivity as SARS-CoV-2 RBD. Two nanobodies, WNb 21 and WNb 23, which displayed no or low-level detection to SARS-CoV-2 RBD, did bind to SARS-CoV RBD (Fig. 1*B*). As expected, an irrelevant nanobody control B12 (directed to a *Plasmodium falciparum* antigen) had no detectable binding to both SARS-CoV-2 and SARS-CoV RBD (44).

To evaluate whether these nanobodies inhibit SARS-CoV-2 RBD-ACE2 complex formation, we developed a fluorescence resonance energy transfer (FRET)-based assay, where ACE2 labeled with DyLight-594 could be shown to interact with RBD labeled with DyLight-488 (*SI Appendix*, Fig. S2). FRET signal in the absence of antibody was designated as 100% FRET signal. The addition of fivefold molar excess of unlabeled SARS-CoV-2 RBD and human ACE2 out-competed the labeled proteins and abolished the signal of the RBD-ACE2 FRET pair (*SI Appendix*, Fig. S2). As expected, because WT SARS-CoV-2 does not use mouse ACE2 as a receptor, unlabeled mouse ACE2 was unable to out-compete the labeled proteins (45). In addition, unlabeled SARS-CoV RBD showed a ∼70% reduction in complex formation, most likely a reflection of its lower affinity interaction with human ACE2 in comparison with SARS-CoV-2 RBD (*SI Appendix*, Fig. S2). Using this assay, we observed that 39 nanobodies blocked RBD-ACE2 complex formation as indicated by a >80% decrease in FRET signal and another five nanobodies showing >50% decrease in FRET signal (Fig. 1*B* and *SI Appendix*, Table S2). The remaining six nanobodies showed no or <50% decrease in FRET signal.

We tested the potential of the 50 nanobodies to neutralize WT SARS-CoV-2 virus (hCoV-19/Australia/VIC01/2020) using a microneutralization assay (MNV) with viral cytopathic effect as a measurement of infectivity (46). Neutralizing activity ranged from 3 to 36,108 nM, with 10 nanobodies (WNb 67, 41, 36, 2, 53, 7, 50, 15, 10, and 19) neutralizing virus at ≤80 nM (Fig. 1*B* and *SI Appendix*, Table S2). These 10 nanobodies also potently abolished RBD-ACE2 complex formation in the FRET assay (Fig. 1*B*). Using bio-layer interferometry, we determined the binding affinities of the panel of nanobodies against SARS-CoV-2 RBD (Fig. 1*C* and *SI Appendix*, Fig. S3 and Table S2). All tested nanobodies bound with high affinities with dissociation constant (*K*_D_) ranging from 0.14 to 19.49 nM (Fig. 1*C*). Collectively, based on their neutralization potencies in MNV assay (<60 nM) and high antibody affinities (*K*_D_ < 0.5 nM), the top four nanobodies were WNb 2, WNb 7, WNb 15, and WNb 36 (Fig. 1*D* and *SI Appendix*, Table S2). WNb 2/WNb 36 and WNb 7/WNb 15 belong to the same clonal lineages (*SI Appendix*, Table S1 and Fig. 1*B*).

To understand whether this collection of nanobodies bound to distinct antigenic sites, we performed antibody competition experiments using at least one representative from each antibody clonal group (Fig. 1*E*). We observed that the nanobodies bound to the RBD in two major groups of competing nanobodies referred to as Cluster 1 and Cluster 2. The two most potent neutralizing nanobodies, WNb 2 and WNb 36 (Cluster 1), competed with each other but did not compete with the majority of WNb nanobodies in Cluster 2.

### Bivalent Nanobody-Fc Fusions Bind to RBD Natural Variants and Neutralize the SARS-CoV-2 D614G N501Y Virus.

The four lead nanobodies were fused to the Fc domain of human IgG1 to allow bivalent binding and prevent rapid clearance in vivo. The resulting WNbFc 2, WNbFc 7, WNbFc 15, and WNbFc 36 fusions purified as dimers and their identity confirmed by mass spectrometry analyses (*SI Appendix*, Fig. S4, PXD023483). The WNbFc fusions retained their ability to bind to distinct antigenic sites on the SARS-CoV-2 RBD, inhibit ACE2-RBD complex formation using the FRET assay, and bind with low nanomolar affinity to SARS-CoV-2 RBD and with picomolar affinity to spike (*SI Appendix*, Fig. S4). Furthermore, using a flow cytometry-based assay, we showed that the four WNbFc fusions blocked the interaction of SARS-CoV-2 spike expressing cells with ACE2 expressing cells in a dose-dependent manner (*SI Appendix*, Fig. S5).

**Fig. 2.**
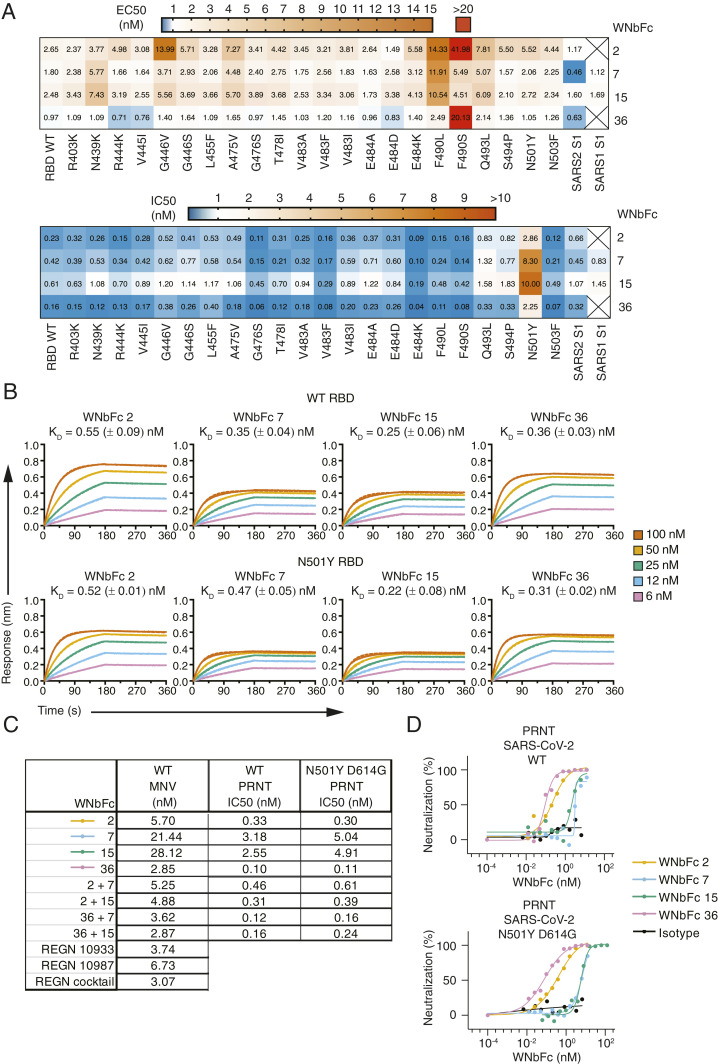
Antibody affinities and neutralization potencies of bivalent nanobody-Fc fusions. (*A*) Heatmap of WNbFc nanobodies binding to RBD variants with the range of relative EC_50_ shown [nanomolar (nM)] (*Top*) and blocking of ACE2 interaction with the range of relative IC50 shown (nM) (*Bottom*) using the RBD global variant multiplex arrays. Squares with crosses represent nonbinders and noninhibitory nanobodies in top and bottom, respectively. (*B*) Bio-layer interferometry affinity measurements with immobilized WNbFc fusion and SARS-CoV-2 RBD in solution. Representative binding curves of five different RBD concentrations from 6 to 100 nM to immobilized nanobodies are plotted (solid line) and were fitted to a 1:1 binding model (dashed line). Corresponding mean ± SEM. *K*_D_ values are indicated, and representative binding curves are shown from three independent experiments. (*C*) Neutralization of WT or N501Y D614G SARS-CoV-2 using the MNV and PRNT assay. For MNV, the values are the geometric mean of *n* = 2 to 4 biological replicates. For PRNT, the value is representative of two biological replicates with duplicate technical replicates per concentration. (*D*) Inhibition of WT and N501Y D614G SARS-CoV-2 virus using PRNT. For PRNT, the value is representative of two biological replicates with duplicate technical replicates per concentration.

We determined the level of binding of WNbFc 2, WNbFc 7, WNbFc 15, and WNbFc 36 fusions to an RBD variant array consisting of the 22 most prevalent RBD natural variants (from RBD surveillance Global Initiative on Sharing Avian Influenza Data (GISAID), June 2020) along with WT SARS-CoV-2 RBD (original Wuhan Strain) and SARS-CoV-2 and SARS-CoV S1 subunit (Fig. 2*A*). These WNbFc 2, WNbFc 7, WNbFc 15, and WNbFc 36 lead candidates bound to most RBD variants with relative half maximal effective concentration (EC_50_) values ranging from 0.7 to 14 nM, with the exception of WNbFc 2 and 36, which had weaker binding to F490S with EC_50_ of 42 and 20 nM, respectively (Fig. 2 *A*, *Top*). WNbFc 2, 7, 15, and 36 bound to WT RBD with relative EC_50_ of 2.65, 1.80, 2.48, and 0.97 nM, respectively, and showed less than 2.5-fold reduction in binding to either E484K or N501Y variant RBDs. Consistent with our initial ELISA results, WNbFc 7 and 15 bound to SARS1 S1, whereas WNbFc 2 and 36 did not (Figs. 1*B* and 2 *A*, *Top*). Using bio-layer interferometry, we further determined the affinities of the WNbFc fusions binding to both WT RBD (Fig. 2 *B*, *Top*) and the N501Y variant (Fig. 2 *B*, *Bottom*) and observed that all bound with *K*_D_ less than 0.55 nM with no significant difference in affinities between either RBD constructs.

Using this RBD variant array, we also examined if the WNbFc fusions were able to block receptor engagement in a multiplex ACE2 competition assay (Fig. 2 *A*, *Bottom*). WNbFc 2, 7, 15, and 36 inhibited ACE2 engagement to WT RBD with relative half maximal inhibitory concentration (IC50s) of 0.23, 0.42, 0.61, and 0.16 nM, respectively, and to the majority of the RBD variants with relative IC50s ranging from 0.04 to 1.8 nM (Fig. 2 *A*, *Bottom*). Notably, all WNbFc fusions exhibited higher relative IC50s with the N501Y variant RBD in comparison to WT but were still able to inhibit ACE2 binding when administered at 10- to 20-fold higher concentrations. The higher IC50 values are most likely a reflection of the higher affinity of human ACE2 to the N501Y variant due to the competitive format of the assay where all binding components (RBD, ACE2, and nanobody) were added simultaneously. All of the WNbFc fusions were able to block ACE2 interaction with the E484K variant with better potencies compared to WT RBD, with relative IC50s of 0.09, 0.10, 0.19, and 0.04 nM for WNbFc 2, 7, 15, and 36, respectively (Fig. 2 *A*, *Bottom*). Furthermore, while WNbFc 2 and 36 showed lower binding to F490S compared to WT RBD, they were still able to inhibit RBD-ACE2 interaction at less than 0.7 nM, suggesting that binding affinities may not fully correlate with the ability to inhibit complex formation. WNbFc 7 and WNbFc 15 were also able to inhibit ACE2 interaction with SARS-CoV S1 subunit at relative IC50 values of 0.83 and 1.45 nM, respectively (Fig. 2 *A*, *Bottom*). WNbFc 36 was the most potent inhibitor of complex formation with most relative IC50s to the RBD variant array within 0.4 nM (with the exception of the N501Y variant RBD).

We compared the ability of these WNbFc fusions, as either single entities or in a two-antibody mixture combination, to neutralize WT SARS-CoV-2 virus using the MNV assay. WNbFc 2, 7, 15, and 36 had neutralizing activity at 5.70, 21.44, 28.12, and 2.85 nΜ, respectively (Fig. 2*C*). The mixture combinations of WNbFc 2 + 7, WNbFc 2 + 15, WNbFc 36 + 7, and WNbFc 36 + 15 had neutralizing activity at 5.25, 4.88, 3.62, and 2.87 nM, respectively (Fig. 2*C*). WNbFc 2 and WNbFc 36 displayed more potent neutralization activity compared to WNbFc 7 and WNbFc 15. In general, the WNbFc fusions, either as single entities or in a mixture combination, had equivalent or better neutralization activity than the previously published REGN 10933 and REGN 10987 antibodies (Fig. 2*C*) (47).

We used a plaque reduction neutralization test (PRNT) to determine if the WNbFc fusions were able to neutralize both the WT (hCoV-19/Australia/VIC01/2020) and SARS-CoV-2 (hCoV-19/Australia/VIC2089/2020) with the D614G N501Y mutations. Genomic sequencing shows that SARS-CoV-2 carrying G614 has replaced D614 as the predominant circulating variant and increases the infectivity of the virus (48, 49). WNbFc 2, 7, 15, and 36 neutralized WT SARS-CoV-2 with IC50 of 0.33, 3.18, 2.55, and 0.10 nM, respectively (Fig. 2 *C* and *D*). In general, the IC50 values for PRNT were ∼5- to 10-fold lower than the neutralizing nanobody titers obtained in the MNV assay, as expected (50). Using SARS-CoV-2 N501Y D614G, WNbFc 2, 7, 15, and 36 showed PRNT IC50 values of 0.30, 5.04, 4.91, and 0.11 nM, respectively, with most showing comparable neutralization potencies to WT virus with less than a twofold difference (Fig. 2 *C* and *D*). The mixture combination WNbFc 2 + 7, WNbFc 2 + 15, WNbFc 36 + 7, and WNbFc 36 + 15 showed PRNT IC50 values ranging between 0.12 to 0.46 nM against WT SARS-CoV-2, with similar neutralization potencies between WT and SARS-CoV-2 N501Y D614G variant (Fig. 2*C*). As in the MNV assay, WNbFc 2 and WNbFc 36 displayed more potent neutralization activity than WNbFc 7 and WNbFc 15 for both WT and variant SARS-CoV-2 viruses. These results show that the WNbFc fusions bound to the N501Y RBD variant at similar antibody affinities compared to WT RBD and neutralized SARS-CoV-2 N501Y D614G virus at comparable potencies to WT SARS-CoV-2, with concentrations between 0.1 to 5.1 nM.

### Structural Characterization of Nanobody Mixtures.

To visualize how the nanobodies bound to SARS-CoV-2 spike protein, we incubated WNb 2 and WNb 10 with the purified ectodomain of SARS-CoV-2 spike containing the six stabilizing proline mutations at a stochiometric ratio of 6:1 for 30 min at room temperature and applied to size-exclusion chromatography (SEC) to isolate the WNb-RBD complex (51). Unfortunately, we were unsuccessful with our attempts with WNb 7 and WNb 15 and instead used WNb 10, as it belongs to the same clonal lineage as WNb 7 and 15, having 93.5% and 95.1% homology, respectively (*SI Appendix*, Table S1 and Fig. S6). Epitope mapping and SEC experiments confirm that WNb 10 bound simultaneously with WNb 2 to SARS-CoV-2 RBD (*SI Appendix*, Fig. S6). Similarly to WNb 7 and 15, WNb 10 also abolished RBD-ACE2 complex formation and neutralized virus at 68.1 nM (Fig. 1*B* and *SI Appendix*, Table S2). Complete details of cryo-EM sample preparation, data processing, and model building are in *SI Appendix*, *Supplementary Materials* and Figs. S7 and S8, and *Materials and Methods*.

**Fig. 3.**
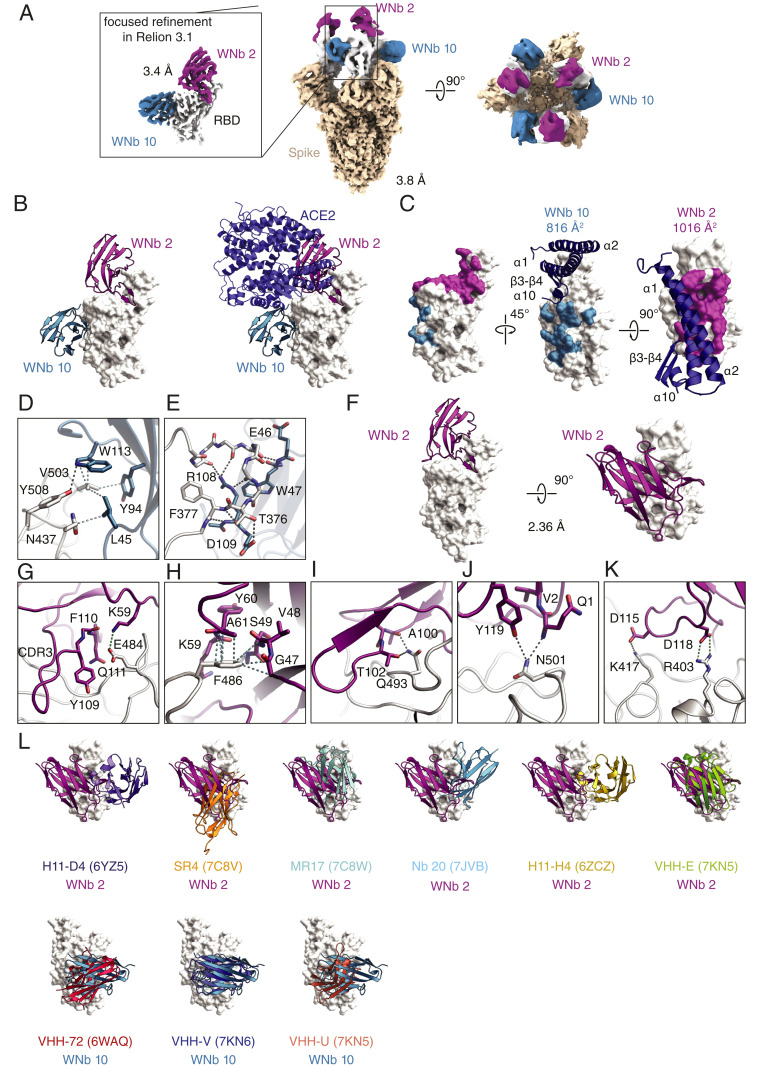
Cryo-EM structure of WNb 2–WNb 10–spike complex and crystal structure of WNb2-RBD complex. (*A*) Cryo-EM maps for spike bound to WNb 2 and WNb 10. (*Middle* and *Right*) Overall map for spike ectodomain with the best resolved densities for six bound WNbs (three of WNb 2 and three of WNb 10) showing all RBDs in the “up” conformation. (*Left*) The map of an individual RBD bound to WNb 2 and WNb 10 following focused 3D classification in Relion 3.1. (*B*) Cryo-EM structure of WNb 2 and WNb 10 bound to the RBD (*Left*) and with human ACE2 superimposed (*Right*). (*C*) The WNb 2 and WNb 10 binding footprint on SARS-CoV-2 RBD are highlighted as magenta and teal, respectively. The overlay of human ACE2 helices is shown in dark blue. (*D* and *E*) Interacting residues between WNb 10 and the RBD. (*F*) Crystal structure of WNb 2-RBD complex. (*G*–*K*) Interacting residues between WNb 2 and the RBD identified from the crystal structure. (*L*) Overlay of existing nanobody structures with WNb 2 and WNb 10. The codes in the brackets refer to the PDB identification codes.

The cryo-EM structure of the complex was solved to an overall resolution of 3.8 Å, with each trimeric spike protein bound by six nanobodies, which represented the binding of both WNb 2 and WNb 10 to individual RBD domains (Fig. 3*A*). Due to the flexibility of the RBDs and some nanobody dissociation, their local resolution is much lower than the overall resolution (<5 Å and <8 Å for RBDs and WNbs, respectively). Focused classification was applied to eliminate spike trimers that were not simultaneously bound to six nanobodies, leading to an overall map at 3.8 Å with slightly improved densities for flexible regions (*SI Appendix*, Fig. S7). We used the model generated from this map to perform a rigid-body fit of RBD-WNb coordinates to understand the overall arrangement of RBD and WNbs in the context of the ectodomain of SARS-CoV-2 spike. All three RBD domains were in the up conformation with no contacts between neighboring RBDs or WNbs. By applying focused classification to individual RBD-WNb complexes, their resolution was improved to 3.4 Å, sufficient for tracing the main chains and placement of most side chain rotamers.

From the cryo-EM structure, WNb 10 binding to RBD did not overlap with WNb 2, which is consistent with the epitope mapping and SEC experiments (Fig. 3*B* and *SI Appendix*, Fig. S6). Binding of both WNb 2 and WNb 10 overlapped with the ACE2 binding site on the RBD (Fig. 3 *B*, *Right*). The buried surface areas at the interface of WNb 2-RBD and WNb 10-RBD were 1016 Å^2^ and 816 Å^2^, respectively (Fig. 3*C*). In comparison, the buried surface area of RBD-ACE2 interface is 870 Å^2^ (Protein Data Bank [PDB] in Europe, proteins, interfaces, structures and assemblies [ePISA], PDB 6M17). Residues 112 to 118 of WNb 10 overlap with the position of the ACE2 α-10 helix when bound to RBD (Fig. 3*C*). This ACE2 α-helix is positioned ∼5 Å from the RBD, with the closest contact between residue Q325 of ACE2 and V503 of the RBD at 4.5 Å. WNb 2 residues 24 to 28, 97 to 100, and 113 to 119 are positioned over the RBD, directly overlapping the binding position of the ACE2 α-1 helix, which contains a large portion of the ACE2 residues responsible for coordinating its interaction with RBD (Fig. 3*C*).

The majority of contacts of WNb 10 with the RBD are mediated by residues 108 and 109 of the complementarity-determining region (CDR) 3 loop, W113, and residues 43 to 47 of framework region (FR) 2. The CDR1 and 2 loops have no contribution to WNb 10 binding to RBD. In general, these WNb 10 residues form two contact sites on the RBD; the first site (site 1) involves RBD residues N437, V503, and Y508 (Fig. 3*D*), and the second site (site 2) involves RBD residues L368 through F377 inclusive (Fig. 3*E*). For the first site, W113 of WNb 10 stacks against V503 and also forms a hydrogen bond with Y508 of the RBD (Fig. 3*D*). Furthermore, WNb 10 residues L45 and Y94 form Van der Waals interactions with V503 and N437 of the RBD. This interaction directly overlaps with the position of ACE2 Q325 when in complex with the RBD. The interactions with RBD at site 1 thus orient WNb 10 in a way that would create steric clashes upon ACE2-RBD binding and inhibit ACE2-RBD complex formation (Fig. 3*C*) (4, 6).

Site 2 is formed between WNb 10 residues R108, D109, E46, and W47 and RBD residues 368 to 377 and does not overlap the ACE2 binding site (Fig. 3*E*). R108 of WNb 10 forms the majority of contacts at this site, with five hydrogen bonds to the RBD. A further two hydrogen bonds are formed between D109 of WNb 10 and T376 of the RBD and between W47 of WNb 10 and A372 of the RBD. Of these, only one hydrogen bond between D109 and T376 involves an RBD amino acid residue sidechain, with the remainder being made to the RBD peptide backbone. WNb 10 does not contact the three RBD residues (417, 484, and 501) mutated in the variants of concern B.1.1.7, B.1.351, and B.1.1.248, and binding should not be impacted by mutations at these positions. The lower relative IC50 values of WNb 10 for the 484K variant (Fig. 2*A*) may be linked to a reduction in ACE2 affinity for RBD that has been described for this variant (52).

We also determined the crystal structure of WNb 2 bound to the SARS-CoV-2 RBD to 2.36 Å resolution (Fig. 3*F* and *SI Appendix*, Table S4). Both of the crystal structures and cryo-EM WNb 2 structures overlap well with an rmsd of 0.796 Å over 2,090 atoms (*SI Appendix*, Fig. S9). One major difference between the cryo-EM and crystal structure of WNb 2 is the alternate conformation seen for F27 of WNb 2 (*SI Appendix*, Fig. S9). In the crystal structure, the positioning of the N-terminal region of WNb 2 differs by 2 Å at the V2 residue; when overlayed, this clashed with the position of the F27 side chain in the cryo-EM structure. This resulted in the adoption of an alternate conformation of F27 in the crystal structure, allowing stacking interactions with T500 of the RBD to form an additional interaction (*SI Appendix*, Fig. S9). The crystal structure of WNb 2 RBD shows that the majority of contacts of WNb 2 with the RBD are mediated through residues within the CDR1 and CDR3 loops and with residues on either side of the CDR2 loops also forming several contacts (*SI Appendix*, Table S5).

A total of 21 RBD residues directly contact WNb 2; of these, E484, F486, Q493, and N501 have all been identified as important for the tighter binding of ACE2 to SARS-CoV-2 over SARS-CoV (15). Residues 484 and 486 are within a stretch of seven amino acids that differ between SARS-CoV and SARS-CoV-2, which results in an altered loop conformation between the two RBDs. E484 forms a salt bridge with K59 of FR3 of WNb 2 (Fig. 3*G*). While the E484K variant lacks the ability to form a salt bridge with K59, our results show that WNb 2 bound to E484A or E484K with relative EC_50_ values of 2 and 5 nM, respectively, and competitively inhibited E484K interaction with ACE2 at higher potency compared to WT RBD (Fig. 2*A*). These results suggest that the salt bridge between E484 of RBD with K59 of WNb 2 may not be crucial for antigen–nanobody recognition.

F486 protrudes from the 481 to 487 loop of SARS-CoV-2 RBD and interacts with ACE2. WNb 2 forms a pocket around F486 and forms more contacts with the F486 sidechain than with any other single SARS-CoV-2 RBD residue (Fig. 3*H*). The F486 sidechain stacks against the peptide backbone of residues 47 to 49 in the CDR2 loop and with residues of 59 to 61 of FR3 as well as the A61 sidechain in FR3 of WNb 2 (Fig. 3*H*).

Q493 on the RBD contacts the WNb 2 CDR3 loop by forming a hydrogen bond between the Q493 sidechain and the backbone carbonyl oxygen of A100 and electrostatic interactions with the T102 sidechain (Fig. 3*I*). N501 on the RBD is important for ACE2 interaction, and the N501Y variant increases binding affinity of the RBD for ACE2. N501 contacts WNb 2 through electrostatic interactions with Q1 and Y119 in a relatively large pocket, which should be able to accommodate larger aromatic residues such as tyrosine (Fig. 3*J*). This is consistent with our results showing that WNb 2 and WNb 36 have similar binding affinities to N501Y compared to WT RBD (Fig. 2*B*).

In addition to the 12 hydrogen bond interactions, there are four salt bridges formed between SARS-CoV-2 RBD and WNb 2 (*SI Appendix*, Table S5). The first two salt bridges are formed between D118 of WNb 2 with the NH1 and NH2 of R403 of the RBD (Fig. 3*K*). D118 is within the CDR3 loop of WNb 2 and conserved in equivalent positions within WNb 36, WNb 41, and WNb 67, which are members of the same clonal lineage (Fig. 1*B* and *SI Appendix*, Table S1 and Fig. S9). The third salt bridge is between D115 of WNb 2 and K417 of RBD (Fig. 3*K*). The K417N variant would disallow the formation of a salt bridge. Interestingly, D115 of WNb 2 is only conserved within WNb 41 but substituted with a Y115 within WNb 36 and WNb 67, which would also disallow salt bridge formation with K417 (*SI Appendix*, Table S1 and Fig. S9). These four nanobodies show equivalent binding affinities to WT RBD, suggesting that the salt bridge between K417 and D115 of WNb 2 is not critical for antigen recognition (*SI Appendix*, Table S2). The fourth salt bridge is between K59 of WNb 2 with E484 on the RBD, which we described earlier as potentially not being crucial for recognition (Fig. 3*G*). Collectively, these results suggested that WNb 2 and WNb 36 will be effective against some of the more prominent global variants in circulation.

The RBD binding sites of published nanobody structures overlap to varying degrees to that of WNb 2 and WNb 10 (Fig. 3*L*). However, none bind in an identical manner, and all five nanobodies show a different binding orientation to RBD compared to WNb 2.

### Prophylactic Administration of WNbFc Fusions Reduce Viral Loads in Mice.

**Fig. 4.**
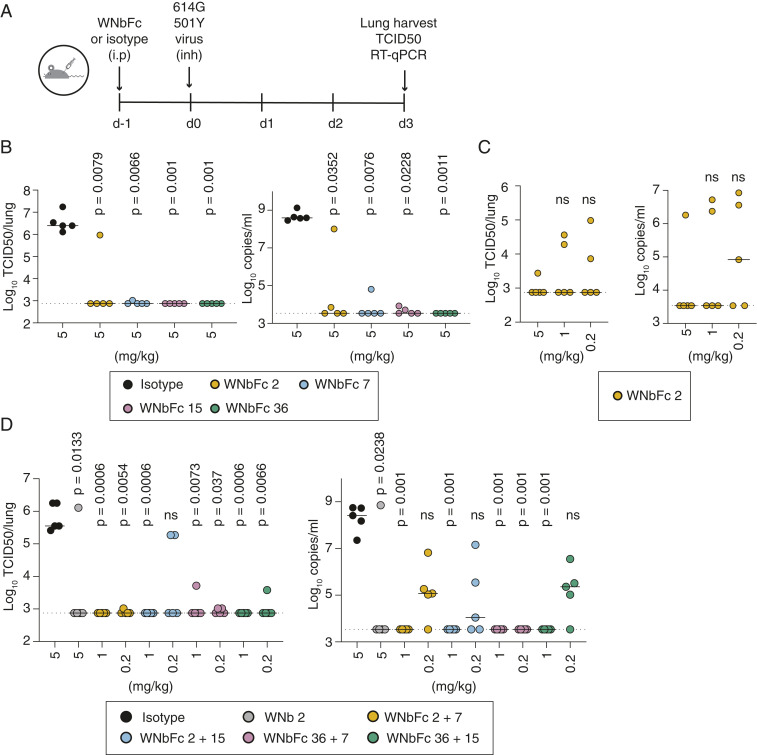
Prophylactic efficacy of neutralizing nanobody-Fc fusions against SARS-CoV-2 infection in mice. (*A*–*D*) Mice were prophylactically administered nanobodies or isotype control via intraperitoneal injection. After 24 h, mice were challenged with SARS-CoV-2 N501Y D614G virus via the Glas-col nebulization inhalation system. Lungs were harvested at day 3 postinfection and homogenized for downstream analyses. (*B*) Mice received 5 mg/kg of the indicated WNbFc fusions. (*C*) Mice received 5, 1, or 0.2 mg/kg WNbFc 2. (*D*) Mice received 1 or 0.2 mg/kg of the indicated WNbFc mixtures, 5 mg/kg of isotype control, or 5 mg/kg of WNbFc 2. (*B*–*D)* Viral burden in the lung was measured by SARS-CoV-2 live virus quantification by TCID50 assay (*Left*) and by RT-qPCR (*Right*). Each circle represents an individual mouse. Dashed lines indicate assay limit of detection. The horizontal lines indicate the median values of animals in each treatment group. To compare TCID50 and RT-qPCR values between groups, a Kruskal–Wallis test across the groups was performed followed with Dunn’s post-test to obtain *P* values comparing the treated groups with isotype controls (*B* and *D*) or highest dose animals (*C*).

To test the prophylactic efficacy of the WNbFc fusions, we administered the antibodies by intraperitoneal injection 24 h prior to challenge of C57BL/6J mice with human clinical isolate of SARS-CoV-2 (hCoV-19/Australia/VIC2089/2020), which has the N501Y D614G mutations (Fig. 4*A*). It has also been previously shown that N501Y significantly increases virus adaptation in a mouse model (23). All animals from the WNbFc 7, WNbFc 15, and WNbFc 36 treatment group and four of five animals from the WNbFc 2 treatment group (5 mg/kg) were protected at 3 d postchallenge, as measured by tissue cultured infectious doses (TCID50) assays of lung tissue homogenates, whereas high levels of virus could be readily detected in isotype control animals (Fig. 4*B*). Each of the WNbFc treatments reduced viral RNA levels up to 10^4^-fold when compared to the isotype control group (Fig. 4*B*). A dose titration of WNbFc 2 from 5, 1 to 0.2 mg/kg, showed lower efficacy of the antibody treatment at lower doses (Fig. 4*C*). Prophylactic administration with the four different WNbFc mixture combinations at 1 mg/kg showed that all combinations suppressed virus infection in either all or four of five mice and reduced viral load up to 10^4^-fold compared to isotype control (Fig. 4*D*). Furthermore, while WNbFc 2 at 5 mg/kg showed protection of 4 of 5 mice, the WNb 2 + 7 and WNb 2 + 15 mixtures at 1 mg/kg protected all mice up to the limit of detection. Using a fivefold lower dose at 0.2 mg/kg, the WNbFc 2 + 7 and WNbFc 36 + 7 mixture combinations were still able to suppress virus in all treated mice to the limit of detection as shown in the TCID50 assay. At the same concentration, WNb 2 + 15 protected three of five mice and WNb 36 + 15 protected four of five mice up to the limit of detection compared to the isotype control in which all mice were infected (Fig. 4*D*). Collectively, these results suggest that the mixture combination of WNb 2 + 7 or WNb 36 + 7 are promising candidates for the prevention of COVID-19.

## Discussion

Here, immunization of alpacas with recombinant spike and RBD proteins enabled the identification of potent neutralizing nanobodies against SARS-CoV-2 and the N501Y D614G variant. This work describes bivalent nanobody-Fc fusions that bound to noncompeting sites on the RBD and blocked ACE2 receptor engagement against a panel of naturally occurring RBD variants. Furthermore, our nanobody-Fc fusions are high affinity (*K*_D_ < 0.55 nM) with potent neutralizing activities against SARS-CoV-2 (as low as 0.1 nM).

The SARS-CoV-2 RBD spans residues 319 to 541 with the RBM spanning residues 438 to 506, which contains most of the contacting residues of SARS-CoV-2 that bind to human ACE2 (6, 15). Since late 2020, three major SARS-CoV-2 variants have emerged: B.1.1.7, B.1.351, and B.1.1.248. Within the RBD, B.1.1.7 harbors the N501Y mutation, whereas B.1.351 has K417N, E484K, and N501Y, and B.1.1.248 has K417T, E484K, and N501Y. Using the RBD variant array in an ACE2 blocking assay, we show that our four WNbFc fusions blocked ACE2 interaction with the E484K RBD variant at a level comparable to WT RBD (Fig. 2). Furthermore, they bound to the N501Y RBD variant with the same antibody affinities compared to WT RBD and, more importantly, neutralized SARS-CoV-2 N501Y D614G virus at comparable potencies to WT SARS-CoV-2 (Fig. 2). From the crystal structure, there is a salt bridge formed between D115 of WNb 2 and K417 of RBD (Fig. 3). The K417N/T variants would disallow the formation of a salt bridge. Furthermore, D115 of WNb 2 is substituted with a Y115 within other clonal lineage members WNb 36 and WNb 67, which would also disallow salt bridge formation with K417, suggesting the salt bridge formation at K417 may not be critical for antigen recognition (*SI Appendix*, Fig. S9). Collectively, these results suggest that the four WNbFc fusions will be effective against some of the more prominent global variants in circulation.

WNb 2/36 and WNb 7/15 separate into two major groups of competing nanobodies referred to as Cluster 1 and Cluster 2, respectively (Fig. 1*E*). Structural characterization of WNb 2 (Cluster 1) and WNb 10 (Cluster 2) revealed nonoverlapping SARS-CoV-2 RBD binding sites (Fig. 3*C*). These RBD binding sites of Cluster 1 and Cluster 2 nanobodies had very different degrees of overlap with the ACE2 binding site (Fig. 3*B*), but both Cluster 1 and Cluster 2 lead candidates were able to block ACE2 interaction with a relative IC50 value <1.0 nM (Fig. 2 *A*, *Bottom*). We propose that steric clashes between WNb 10 and ACE2, away from the RBD surface, might contribute to ACE2 blocking despite the relatively small binding overlap on the RBD.

Five crystal structures of neutralizing nanobodies show a different binding orientation to RBD compared to WNb 2. Sybody SR4 (PDB 7C8V) binds a similar surface on the RBD but has its CDR3 loop on the opposite side of the ACE2 binding surface (42). The H11-D4 nanobody (PDB 6YZ5) binds to the side of the RBD across from the ACE2 binding site and partly overlaps the WNb 2 CDR3 loop (37). The H11-H4 (PDB 6ZCZ) nanobody adopts the same positioning over the RBD as H11-D4, and the CDR loops adopt very similar conformations (37). Sybody MR17 (PDB 7C8W) binds a similar overlapping epitope on the ACE2-RBD binding interface though, with the CDR1 loop of MR17 binding the RBD at a position equivalent to residues 103 to 106 of the WNb2 CDR3. The CDR3 loop of MR17 is positioned over the RBD, similarly to the WNb 2 FR4 region (42). Finally, Nb20 (PDB 7JVB) overlaps with approximately half of the WNb 2 binding site but also binds in a different orientation (32). A sixth nanobody, VHH-E, overlaps the position of WNb 2 with the CDR loops placed in similar positions over the RBD surface and contacts mediated mainly through the CDR1 and CDR3 loops (40). In comparison to the WNb 2-RBD interface, the buried surface area of the VHH-E-RBD interaction is lower at 820.7 Å2, and the CDR3 loop of VHH-E is raised away from the RBD surface at residues D110 to W115. This leads to a lack of direct contacts mediated by this part of the CDR3 loop, and residues 403, 417, and 484, which form salt bridges with WNb 2, are not directly contacted by VHH-E. The WNb 2-RBD interface has the largest buried surface area in comparison to the buried surface areas ranging from 599 Å^2^ (H11-D4) to 895.2 Å^2^ (MR17) for the other nanobodies (37, 42). WNb 2 formed more hydrogen bond interactions with the RBD compared to the six overlapping structures, with 16 bonds (of which 4 are salt bridges) in WNb 2 compared with 13 in Sybody MR17 and only 6 in Sybody SR4.

The landscape of initial human antibody generation to SARS-CoV-2 RBD has been extensively mapped during the course of the pandemic (11, 53–59), defining several preferred immunoglobulin heavy chain variable region (IGHV) gene usages and public clonotypes as well as rarer individual epitopes. Preferred gene usages include IGHV3-53/66, IGHV 1 to 2, and IGHV1-58*01 paired with IGKV3-20*01 (53–55). None of the currently defined antibody epitopes overlay completely with that of WNb 2 on the RBD surface; however, there are both public and individual antibodies that share partial overlap with the WNb 2 binding site.

IGHV3-53/66 antibodies bind in one of two modes on the RBD, defined as binding mode 1 and 2 (56). Binding mode 1 antibodies, including B38 (60), recognize residues including K417 and Y489; some additionally contact N501 but do not interact with E484 or the WNb 2 contact regions 351 and 446 to 449. Binding mode 2 antibodies are positioned over the E484 residue but do not bind at K417 or N501, which form a salt bridge and electrostatic interactions to WNb2, respectively (61). IGHV1-2 antibodies are similarly positioned over the E484 residue (13, 14) with some variation in footprint due to differing CDR3 length and light chain usage (62). The IGHV1-58*01 and IGKV3-20*01 antibodies overlap WNb 2 in the 482 to 486 region and around residues L452 and Q493 (57, 63). Individual responses that partly overlap the WNb binding site include P2B-2F6 (VH4-38, PDB: 7BWJ), 1 to 57 (VH3-72, PDB: 7LS9), C104 (VH4-34, PDB: 7K8U), and C110 (VH5-51, PDB: 7K8V), which overlap around residue Y449 and the 490 to 494 region (14, 58, 64).

VHH-72 (PDB 6WAQ), which recognizes both SARS-CoV-2 and SARS-CoV RBD, overlaps with the binding site of WNb 10 (38). The buried surface interface with the RBD is similar to that of WNb 10 at 830 Å^2^ and overlaps the CDR3 of WNb 10 at residues 97 to 103 and 108 to 111. However, VHH-72 lacks the WNb 10 interactions with the RBD, which are mediated by residues W113 and 43 to 47, and therefore does not fully overlap the WNb 10 RBD epitope (*SI Appendix*, Table S5). VHH-V overlaps the RBD epitope bound by WNb 10 and has a larger buried surface area at the interface of 992.3 Å^2^ (40). Contacts are mediated through the VHH-V CDR3 loop and FR2 region, with more interactions with RBD residue sidechains across residues 369 to 380 in comparison to WNb 10. VHH-U partly overlaps the RBD epitope bound by WNb 10, though with a smaller buried surface area at the interface of 625.4 Å^2^ (40). The majority of contacts are formed by the CDR3 loop with less involvement of FRs in comparison to WNb 10. The VHH-U epitope does not include residues 437 to 440 of the RBD, which are bound by FR2 residues in WNb 10. To the best of our knowledge, these structural comparisons suggested that WNb 2 and WNb 10 may bind to SARS-COV-2 RBD using additional interactions not present in other neutralizing nanobodies to date.

Neutralizing antibodies against SARS-CoV-2 are an important therapeutic option against COVID-19. Nanobody and antibody mixtures targeting nonoverlapping epitopes on the RBD have shown promise in preventing occurrence of resistance mutations (7, 40). In particular, a biparatopic nanobody construct capable of simultaneously binding nonoverlapping RBD epitopes showed a 12-time improvement in pseudotyped virus neutralization and prevented occurrence of resistance mutations (40). These neutralizing nanobody-Fc fusions may find an application for the passive immunization of people who are immunocompromised and may not respond as well to vaccination or for preventing outbreaks in high-risk settings, such as aged care facilities. The use of nanobody mixtures may be advantageous for controlling more highly infectious variants and reducing the potential for virus escape mutations to develop.

## Materials and Methods

### Isolation of SARS-CoV-2 Spike and RBD Nanobodies.

Biopanning for SARS-CoV-2 spike and RBD nanobodies using phage display was performed as previously described with the following modifications (28). Phages displaying SARS-CoV-2 spike and RBD-specific nanobodies were enriched after two rounds of biopanning on 10 μg/mL (or 1 μg/well) of either immobilized SARS-CoV-2 spike or RBD protein. After the second round of panning, individual clones were selected for further analyses by ELISA for the presence of SARS-CoV-2 spike and RBD nanobodies, respectively. Positive clones were sequenced and annotated using the International ImMunoGeneTics database and aligned in Geneious Prime (65).

### Antibody Specificity ELISA.

The 96-well flat-bottomed MaxiSorp plates were coated with 125 nM of recombinant protein, as indicated, in 50 μL DPBS at room temperature for 1 h. All washes were done three times using PBS and 0.1% Tween, and all incubations were performed for 1 h at room temperature. Coated plates were washed and blocked by incubation with 10% skim milk solution. Plates were washed and then incubated with 125 nM of nanobodies. The plates were washed and incubated with mouse anti-His (Bio-Rad MCA-1396; 1:1,000) followed by horseradish peroxidase–conjugated goat anti-mouse secondary antibody (MerckMillipore AP124P, 1:1,000). After a final wash, 50 μL azino-bis-3-ethylbenthiazoline-6-sulfonic acid (liquid substrate; Sigma) was added and incubated in the dark at room temperature, and 50 μL 1% SDS was used to stop the reaction. Absorbance was read at 405 nm, and all samples were done in duplicate.

### FRET Assay.

SARS-CoV RBD and SARS-CoV-2 RBD were labeled with *N*-hydroxysuccinimide ester-activated DyLight 488 (DL488) and ACE2 labeled with DyLight 594 (DL594) (Life Technologies). The dyes were dissolved in dimethyl sulfoxide (Sigma) and added at a fivefold molar excess to the protein being labeled. After 1 h incubation at room temperature, unconjugated dye was removed by buffer exchange using a 10 kDa molecular weight cutoff Amicon Ultra-4 centrifugal unit (Merck). Average dye per protein was ∼2.1 dye/protein for SARS-CoV RBD, ∼1.9 dye/protein for SARS-CoV-2 RBD, and ∼1.5 dye/protein for ACE2. In the competition experiment with unlabeled proteins, ACE2-DL594 was mixed with either SARS-CoV RBD-DL488 or SARS-CoV-2 RBD-DL488 in a 1:1 molar ratio with a fivefold excess of unlabeled SARS-CoV RBD, SARS-CoV-2 RBD, human ACE2, or mouse ACE2 in a final reaction volume of 10 μL in FRET buffer (50 mM Tris HCl pH 6.8, 150 mM NaCl). To test inhibitory nanobodies, SARS-CoV-2 RBD-DL488 and ACE2-DL594 were mixed in a 1:1 molar ratio with a fivefold excess of nanobody. The FRET assays were read in Corning 384-well plates, and each sample was performed in triplicate. A total of 1 μL 1% SDS was added to one triplicate sample to measure the background signal. Fluorescence intensity was measured using EnVision plate reader (PerkinElmer Life Sciences). DL488 (donor) fluorescence was measured with a 485/14 nm excitation filter and a 535/25 nm emission filter, and DL594 (acceptor) fluorescence was measured with a 590/20 nm excitation filter and a 615/9 nm emission filter. Sensitized emission was measured with a 485/14 nm excitation filter and a 615/9 nm emission filter. Fluorescence measures were analyzed using Prism 6 software (GraphPad). To account for bleed-through of dyes into the sensitized emission spectra, standard curves for both dyes were measured, and the raw data were transformed by multiplying with the slope of the standards. The *y*-intercept from the standard curves indicated the background fluorescence when no dye was present. The FRET signal was calculated with the following equation: FRET signal = raw sensitized emission − transformed DL488 emission − transformed DL594 emission − (average of DL488 standard curve *y*-intercept and DL594 standard curve *y*-intercept). To compare between biological replicates, percentage FRET signal relative to the no inhibitor control was calculated by dividing the FRET signal with inhibitor by the FRET signal with no inhibitor, multiplied by 100.

### Bio-Layer Interferometry.

Nanobody affinities to SARS-CoV-2 RBD were measured using an Octet RED96 instrument (ForteBio). Assays were performed at 25 °C in solid black 96-well plates agitated at 1,000 rpm. Kinetic buffer was composed of PBS 0.1% BSA, 0.05% TWEEN. A 60 s biosensor baseline step was applied before nanobodies were loaded onto Ni-NTA sensors (FortéBio) by submerging sensor tips in 5 μg/mL of each nanobody until a response of 0.5 nm was obtained and then washed in kinetic buffer for 60 s. Association measurements were performed using a twofold concentration gradient of SARS-CoV-2 RBD from 6 to 200 nM for 180 s, and dissociation was measured in kinetic buffer for 180 s. Sensor tips were regenerated using a cycle of 5 s in 300 mM imidazole pH 7.5 and 5 s in kinetic buffer, repeated five times. Baseline drift was corrected by subtracting the shift of a nanobody-loaded sensor not incubated with SARS-CoV-2 RBD. Curve fitting analysis was performed with Octet Data Analysis 10.0 software (ForteBio) using a global fit 1:1 model to determine *K*_D_ values and kinetic parameters. Curves that could not be fitted were excluded from the analysis.

WNbFc antibody affinities to SARS-CoV-2 RBD, SARS-CoV-2 spike, SARS-CoV-2 RBD WT, and SARS-CoV-2 RBD N501Y were measured using the above method with the following modifications. Anti-human IgG Fc capture sensor tips were used for affinity measurements. For measuring affinities against SARS-CoV-2 RBD, SARS-CoV-2 RBD WT, and SARS-CoV-2 RBD N501Y, WNbFc antibodies were loaded onto sensor tips by submerging in 5 μg/mL WNbFc antibody for 200 s. For measuring affinity against SARS-CoV-2 spike, WNbFc antibodies were loaded onto sensor tips by submerging in 5 μg/mL WNbFc antibody until a signal shift of 0.5 nm.

### Epitope Binning Using Bio-Layer Interferometry.

In WNb competition experiments using bio-layer interferometry, 50 nM SARS-CoV-2 RBD was preincubated with each nanobody at a 10-fold molar excess for 1 h at room temperature (RT). A 30 s baseline step was established between each step of the assay. NTA sensors were first loaded with 10 μg/mL nanobody for 5 min. The sensor surface was then quenched by dipping into 10 μg/mL of an irrelevant nanobody for 5 min. Nanobody-loaded sensors were then dipped into premixed solutions of SARS-CoV-2 RBD and nanobody for 5 min. Nanobody-loaded sensors were also dipped into SARS-CoV-2 RBD alone to determine the level of SARS-CoV-2 RBD binding to immobilized nanobody in the absence of other nanobodies. Percentage competition was calculated by dividing the maximum response of the premixed SARS-CoV-2 RBD and nanobody solution binding by the maximum response of SARS-CoV-2 RBD binding alone, multiplied by 100.

In WNbFc competition experiments using bio-layer interferometry, NTA sensors were loaded with 3 μg/mL SARS-CoV-2 RBD for 5 min. After loading, sensor tips were first submerged into wells containing 200 nM WNb-Fc for 10 min and then subsequently dipped into wells containing 200 nM of a second WNbFc for 5 min. One SARS-CoV-2 RBD loaded sensor was dipped only into the second set of WNbFc antibodies to determine the maximum response in the absence of the first WNbFc antibody binding. Competition was calculated by dividing the maximum response of the second WNbFc binding to immobilized SARS-CoV-2 RBD in the presence of the first WNbFc by the maximum response of the second WNbFc binding in the absence of the first WNbFc.

### Cryo-EM Sample Preparation and Data Acquisition.

#### Sample preparation.

The complexes of spike trimer bound to WNb 2 and WNb 10 were purified over SEC and concentrated to 2.5 mg/mL. The grids (Quantifoil R1.2/1.3 Cu/Rh 200 mesh) were glow discharged in air at 30 mA for 30 s using Pelco EasyGlow. The 3 µL samples were applied to the grids at 4 °C and 100% humidity and plunge frozen in liquid ethane using Vitrobot Mark IV (Thermo Fisher).

Data were collected on Titan Krios (Thermo Fisher) 300 kV electron microscope using K2 detector (Gatan). The acquisition was performed at 165 K indicated magnification using the energy filter slit width of 15 eV. The data were collected during two sessions. For the first session, the 4,154 movies with 56 frames were collected at 0.86 Å pixel size, with a total exposure of 55.9 e/Å^2^. For the second session, the 4,114 movies with 56 frames were collected at 1.06 Å pixel size, with a total exposure of 55.9 e/Å^2^. The data were collected using FEI EPU software using a nine-hole beam-image shift acquisition scheme with one exposure in each hole.

#### Data processing.

The two datasets were initially processed independently. Movies from each of the imaging sessions were subjected to the correction of beam-induced motion using MotionCor2 (66), followed by contrast transfer function (CTF) estimation using Gctf (67). The 3,807 and 3,828 micrographs, from sessions 1 and 2, respectively, with CTF fit resolution below 4 Å, were selected for further examination. Initial particle picking was performed using Cryolo. Particle coordinates were imported into Relion 3.1 (68), extracted and subjected to two-dimensional (2D) classification. Well-defined classes were used for template-based autopicking in Relion (560,825 and 908,985 particles from datasets 1 and 2, respectively). After extraction (416 pixels, 4× binned to 104 pixels), particles were imported into the CryoSparc (69) for 2D classification and multiples rounds of three-dimensional (3D) classifications. The homogeneous set of particles was imported back into Relion 3.1 using the PyEM software package (Asarnow, D., Palovcak, E., and Cheng, Y. UCSF pyem v0.5. Zenodo) and subjected to another round of 2D classification. Particles were reextracted using 416 pixel box size and subjected to another round of 3D classification. At this point, the particles from both datasets (65,166 and 92,058 particles from datasets 1 and 2, respectively) were merged together and used for a consensus refinement of spike bound to WNb 2 and WNb 10.

Merged particles were subjected to multiple rounds of CTF Refinement (beam tilt, trefoil, fourth order aberrations, anisotropic magnification, and per-particle defocus and astigmatism) and Bayesian Polishing (70), followed by another round of 2D classification. Following 3D refinement, the final dataset of 152,752 particles yielded the 3.2 Å map (C1 symmetry) or 2.9 Å map (C3 symmetry) (“best spike” map) based on the gold standard Fourier shell correlation cutoff of 0.143.

To improve the density of the RBD domains bound to nanobodies, the C1 “best spike” map was subjected to a focused classification. The density outside of the RBD domains and nanobodies was subtracted, and the new particle stack was subjected to multiple rounds of masked 3D classification without alignments. All RBD appeared to be in the “up” position; however, the nanobody density appeared variable, either due to the flexibility of the RBD domains or due to nanobody dissociation. The classes representing particles with the most intact nanobodies were selected and unsubtracted. The final dataset of 22,923 particles yielded the 3.76 Å map (C1 symmetry) with the best resolved RBDs and Nbs relative to the spike (“best overall” map).

To get the higher resolution of the RBD interface with WNb 2 and WNb 10, we performed focused classification on each individual RBD domain bound to its respective nanobodies. The density for everything except each individual RBD-WNb complex was subtracted (three times for each RBD-WNb complex) following by 3D classifications without alignment to identify the most intact RBD-WNb complexes (“good” RBD-WNb). Due to the small size of RBD-WNb complexes, we initially aligned all “good” RBD-WNb complexes onto each other using the full spike protein as a handle. Following signal unsubtraction from RBD-WNb complexes, each set of particles was subjected to another round of signal subtraction leaving only the signal for spike bound to a single “good” RBD-WNb. The 3D refinement aligned all remaining RBD-WNb complexes onto each other. This was followed by signal subtraction of the spike leaving only a single RBD-WNb complex, which was followed by two rounds of 3D classification and 3D refinement using local searches and tight mask around the RBD and WNbs. The final dataset of 151,630 particles yielded the 3.44 Å map (“best RBD+WNb2+WNb 10” map).

Local resolution was determined using the internal local resolution procedure in Relion, using half-reconstructions as input maps.

#### Model building.

For the “best RBD+WNb 2+WNb 10” map, the high-resolution model of RBD-WNb 2 from the crystal structure was used for rigid-body docking of the RBD, WNb2, and WNb 10, followed by iterative model adjustment, rebuilding in COOT (71), and real-space refinement in PHENIX (72). For the “best overall” map, previously deposited coordinates of the spike hexapro (PDB 6XKL) (without the RBD domain) and the refined structure of the RBD bound to WNb 2 and WNb 10 were used for rigid-body docking into the cryo-EM map. Model validation was performed in MolProbity (73). Figures were prepared and molecular graphics and analyses performed with UCSF ChimeraX (74) or PyMOL Molecular Graphics System, Version 2.0 (Schrödinger, LLC).

### WNb 2 Crystallography and Structure Determination.

Crystallization trials were undertaken at the Collaborative Crystallization Centre at Commonwealth Scientific and Industrial Research Organisation at 20 °C using 96-well sitting drop vapor diffusion plates (Greiner). The SARS-CoV-2 RBD-WNb 2 complex was set up at 10 mg/mL, and crystals first appeared in a solution containing 20% polyethylene glycol (PEG) 3350 and 0.2 M potassium iodide. Diffraction quality crystals were obtained after 21 d from a solution containing 15% PEG 4,000, 0.2 M potassium thiocyanate, and 0.05 M sodium cacodylate pH 6.0, seeded from the initial condition. Crystals were flash-frozen in liquid nitrogen at 100 K following equilibration in a solution of mother liquor containing 35% PEG 4,000 as a cryoprotectant. A dataset for the SARS-CoV-2 RBD-WNb 2 complex at 2.36 Å was collected on the MX2 beamline at the Australian Synchrotron. The data were recorded using an Eiger 16M detector (Dectris) and processed using the XDS package (75). Phaser was used for molecular replacement using a previously determined nanobody structure (PDB ID 5TP3) and SARS-CoV-2 RBD (PDB identification code [ID] 6W41, chain C) as search models. The WNb 2-RBD complex consisted of four copies of the complex in the asymmetric unit. Refinement of the complex was undertaken through iterative rounds of model building in COOT (71) and refinement in Phenix (76), with fourfold noncrystallographic symmetry restraints applied throughout. Figures were prepared using the PyMOL Molecular Graphics System, Version 2.3.0 (Schrödinger, LLC). Interfaces and interactions were analyzed and buried surface areas calculated using PISA (77). The SARS-CoV-2 RBD-WNb 2 structure has been deposited in the PDB under accession code 7LDJ.

### Prophylaxis Studies in Mice Using SARS-CoV-2 D614G N501Y Virus.

C57BL/6J mice were bred and housed at the Walter and Eliza Hall Institute of Medical Research. All procedures involving animals and live SARS-CoV-2 were conducted in an Office of Gene Technology Regulator-approved Physical Containment Level 3 facility at the Walter and Eliza Hall Institute of Medical Research (Cert-3621; IA88_20). All animal procedures were approved by the Walter and Eliza Hall Institute of Medical Research Animal Ethics Committee (2020.016). Nanobodies were administered to mice in 100 μL PBS by intraperitoneal injection 24 h prior to infection. SARS-CoV-2 infection (clinical isolate hCoV-19/Australia/VIC2089/2020) of C57BL/6J mice was performed using an inhalation exposure system (Glas-Col, LLC) for 45 min loaded with 1.5 × 10^7^ SARS-CoV-2 TCID50. Mice used for experimentation were 7 to 10 wk of age.

## Supplementary Material

Supplementary File

## Data Availability

Cryo-EM maps for WNb 2 WNb 10 SARS-CoV-2 Spike complex are available in the the Electron Microscopy Data Bank (EMDB) (EMD-23566). The WNb 2 WNb 10 SARS-CoV-2 RBD co-ordinates are available in the Protein Data Bank (PDB) (7LX5). The WNb 2 SARS-CoV-2 RBD crystallography data are available in the Protein Data Bank (PDB) (7LDJ). All other study data are included in the article and/or *SI Appendix*.
