# Supplementary material for: Nanobody cocktails potently neutralize SARS-CoV-2 D614G N501Y variant and protect mice
Source: Proc Natl Acad Sci U S A. 2021 Apr 23;118(19):e2101918118. doi: 10.1073/pnas.2101918118 (PMC8126837; doi:10.1073/pnas.2101918118)
Supplement: Supplementary File [file pnas.2101918118.sapp.pdf]

PNAS
www.pnas.org

Supplementary Information for

Nanobody cocktails potently neutralize SARS-CoV-2 D614G N501Y variant and protect mice

Phillip Pymm[1,2#], Amy Adair[1#], Li-Jin Chan[1,2†], James P. Cooney[1,2†], Francesca L. Mordant[3], Cody C. Allison[1,2], Ester Lopez[3], Ebene R. Haycroft[3], Matthew T. O'Neill[1], Li Lynn Tan[1], Melanie H. Dietrich[1,2], Damien Drew[1], Marcel Doerflinger[1,2], Michael A. Dengler[1,2], Nichollas E. Scott[3], Adam K. Wheatley[3,4], Nicholas A. Gherardin[3,5], Hariprasad Venugopal[6], Deborah Cromer[7,8], Miles P. Davenport[7], Raelene Pickering[9], Dale I. Godfrey[3,5], Damian F.J. Purcell[3], Stephen J. Kent[3,4], Amy W. Chung[3], Kanta Subbarao[3,10], Marc Pellegrini[1,2], Alisa Glukhova[1,11,12], Wai-Hong Tham[1,2,*]

Corresponding author: Wai-Hong Tham
Email: tham@wehi.edu.au

This PDF file includes:

Supplementary text
Figures S1 to S9
Tables S1 to S5
SI References

Supplementary Information Text

Extended Methods

Alpaca immunisation

Two alpacas were used for this immunization campaign. The first alpaca was subcutaneously immunized with approximately 50 to 200 mg of recombinant SARS-CoV-2 spike and SARS-CoV-2 RBD proteins, the second with SARS-CoV-2 spike, SARS-CoV RBD and SARS-CoV-2 RBD proteins on days 0, 14, 21, 28, 35 and 42. The adjuvant used was GERBU FAMA. Immunization and handling of the alpacas for scientific purposes was approved by Agriculture Victoria, Wildlife & Small Institutions Animal Ethics Committee, project approval No. 26-17. Blood was collected three days after the last immunization for the preparation of lymphocytes. Nanobody library construction was carried out according to established methods as described (1). Briefly, alpaca lymphocyte mRNA was extracted and amplified by RT-PCR with specific primers to generate a cDNA library size of 10^8 nanobodies with 80% correct sized nanobody inserts. The library was cloned into a pMES4 phagemid vector amplified in *E. coli* TG1 strain and subsequently infected with M13K07 helper phage for recombinant phage expression.

Recombinant protein purification for spike, RBD and ACE2 proteins

For immunization of alpacas and in vitro assays, two different SARS-CoV-2 RBD constructs with a C-terminal His-tag (residues 319-541 and residues 331-527) were expressed in Expi293 cells and purified by Ni-NTA followed by size-exclusion chromatography (SEC) using a Hiload 16/600 Superdex 75 pg gel filtration column (GE Healthcare) (2). SARS-CoV RBD (residues 321-513) with a C-terminal AviTag and His-tag, was expressed in Expi293 cells and purified by nickel-nitrilotriacetic acid agarose (Ni-NTA) resin columns followed by SEC using a Superdex 75 increase 10/300 GL (GE Healthcare). The ectodomain of SARS-CoV-2 (isolate WHU1; residues 1 – 1208) was synthesised with furin cleavage site removed and P986/987 stabilisation mutations, a C-terminal T4 trimerisation domain, AviTag and His-tag and expressed and purified as previously described (3, 4). The human (residues 19-612) and mouse (residues 19-615) ACE2 ectodomain with C-terminal His-tag were expressed in Expi293 cells and purified using Ni-NTA and SEC.

Nanobody expression and purification

Nanobodies were expressed in *Escherichia coli* WK6 cells. Bacteria were grown in Terrific Broth at 37 °C to an optical density at 600 nM (OD_{600}) of 0.7, induced with 1 mM isopropyl β- d-1-thiogalactopyranoside (IPTG) and grown overnight at 28 °C for 16 h. Cell pellets were harvested and resuspended in 20% sucrose, 20 mM imidazole, 150 mM NaCl Dulbecco's phosphate buffered saline (DPBS) and incubated for 15 min on ice. 5 mM ethylenediaminetetraacetic acid (EDTA) was added and incubated on ice for 20 minutes. After this incubation, 10 mM $MgCl_2$ was added to prevent nickel ion-EDTA chelation, periplasmic extracts were harvested by centrifugation and the supernatant was loaded onto a 1 ml HisTrap Fast Flow (FF) column (GE Healthcare). The nanobody was eluted with 400 mM imidazole, 100 mM NaCl, DPBS. The appropriate fractions were concentrated and buffer exchanged in to sterile DPBS.

WNbFc fusion cloning, expression and purification

Synthetic human IgG1-Fc sequence (IDT) was cloned into the pHLSec vector via restriction sites AgeI and XhoI to generate pHLSec-Fc. Nanobody-encoding sequences were amplified by PCR using Nano15-fwd (5'- TAGACCGGTCAGGTGCAGCTGCAG-3') and Nano18-rev, (5'- CTAGCTAGCGAGGGGACGGTCACCTGG-3') and inserted in pHLSec-Fc via AgeI and NheI to generate the respective WNbFc expression constructs. Recombinant WNbFc fusions were expressed in Expi293 HEK cells (ThermoFisher), which were maintained in suspension at 37°C and 8% CO_2. Cells were transfected at a density of 3 x 10^6 with 1 μg of plasmid DNA for 1 mL of culture and ExpiFectamine™ 293 reagent diluted in Opti-Mem™ (ThermoFisher) following the manufacturer's protocol. 22 hours after transfection, ExpiFectamine™ 293 transfection Enhancer 1 and 2 (ThermoFisher) was added to transfected cells along with lupin peptone. Five days after transfection, the supernatant was collected by centrifugation and filtered through a 0.22 μm filter. WNbFcs were purified by loading the supernatant onto a 1 mL Protein A HP HiTrap column (GE Healthcare). Columns were equilibrated and washed using DPBS. WNbFcs were eluted using 0.1 mM citric acid pH 3.0 and neutralized with 1M Tris-HCl pH 9.0. A second purification step was performed by loading Protein A eluate on a Hiload 16/600 Superdex 200 pg gel filtration column (GE Healthcare), which was equilibrated and run using DPBS. WNbFcs were concentrated using Amicon Ultra-4 30 kDa (Millipore).

Antibody concentration was determined by absorbance measurement at 280 nm using a Nanodrop and purity was determined using sodium dodecyl-sulfate polyacrylamide gel electrophoresis (SDS-PAGE).

Microneutralization assay

SARS-CoV-2 isolate hCoV/Australia/VIC01/2020 was passaged in Vero cells and stored at −80 °C. The ability of nanobodies to neutralize the infectivity of 100 median tissue culture infectious doses (TCID50) of virus was assessed in a microneutralization assay as previously described (4) . Serial two-fold dilutions of nanobodies starting at 1:20 were incubated with SARS-CoV-2 in minimum essential media (MEM)/0.5% bovine serum albumin (BSA) at room temperature for 1 h. Residual virus infectivity was assessed in quadruplicate wells of Vero cells and viral cytopathic effect was read on day five. The neutralizing antibody titer was calculated using the Reed–Muench method as previously described (4).

RBD global variant multiplex arrays

An RBD variant array consisting of 23 SARS-CoV-2 RBD variants including the wildtype (WT) isolate WHU1 were selected from the GISAID RBD surveillance repository (June 2020). SARS-CoV2 RBD sequence (GenBank: MN908947.3. (amino acids 319–541; RVQP…CVNF), along with the signal peptide (amino acids 1–14; MFVF…VSSQ) was used as the original WT sequence) plus a hexahistidine tag were cloned into pcDNA3 vectors by Genscript Corporation (Piscataway, NJ, USA). SARS-CoV-2 S1 (#40591-V08H, Sino Biologicals) and SARS-CoV S1 (#S1N-S52H5, AcroBiosystems) recombinant proteins were also included in the array.

RBD variants were expressed in Expi293 HEK cells (ThermoFisher), which were maintained in suspension at 37 °C and 8% CO_2. Cells were transfected at a density of 3 x 10^6 with 1 µg of plasmid DNA for 1 mL of culture and ExpiFectamine™ 293 reagent diluted in Opti-Mem™ (ThermoFisher) following the manufacturer's protocol. 22 hours after transfection, ExpiFectamine™ 293 transfection Enhancer 1 and 2 (ThermoFisher) was added to transfected cells along with lupin peptone. Five days after transfection, the supernatant was collected by centrifugation, 10 mM $MgCl_2$ was added to improve binding to the column and filtered through a 0.22 µm filter. RBD variants were purified by loading the supernatant onto a 1 mL Ni Excel column (GE Healthcare). Columns were equilibrated

and washed using DPBS. RBD variants were eluted using 300 mM imidazole, 100 mM NaCl DPBS buffer. A second purification step was performed by loading the eluate on a Superdex75 increase 10/300 GL gel filtration column (GE Healthcare), which was equilibrated with DPBS. Recombinant proteins were concentrated using Amicon Ultra-4, 10 kDa (Millipore). Protein concentration was determined by absorbance measurement at 280 nm using a Nanodrop and purity was determined using SDS-PAGE.

RBD variant multiplex bead cocktails was generated as previously described (5). Briefly, each respective RBD protein was coupled to a different magnetic carboxylated bead region (Bio Rad) using a two-step carbodiimide reaction, at a ratio of 1 million beads to 5 μg of each RBD antigen.

The experiment was conducted in black, clear bottom 384-well plate (Greiner Bio-One). RBD global variant cocktails (1000 beads of each bead region per well) were added to nanobodies at final concentration of 80 nM per well and subsequent 8-fold 1:4 titrations. Plates were incubated for two hours on a plate shaker at RT, washed twice in 0.05% PBS Tween 20, and relative nanobody binding was detected using anti-human IgG R-Phycoerythrin (PE) Conjugate (#9040-09, Southern Biotech) at 1.3 μg/ml for two hours RT with shaking. Plates were then washed three times, sheath buffer was added to each well and plates were acquired on a FlexMap3D™ (Luminex Corporation). The binding of IgG-PE to each bead was detected as MFI (Median Fluorescence Intensity).

RBD ACE2 inhibition assays were conducted using RBD variant multiplex bead cocktails (1000 beads of each bead region per well) in black, clear bottom 384-well plates. 20 μl of 25 μg/ml of Avi-Tagged biotinylated ACE2 along with 10 μl of nanobodies (final concentration of 80 nM per well, with subsequent 8-fold 1:4 titrations). Human ACE2 (residues 19–613) ectodomain with a C-terminal His-tag was expressed in Expi293 cells and purified using Ni-NTA and size-exclusion chromatography before being biotinylated using Bir-A (Avidity). Total final volume per well was 50 μl. Plates were then incubated for 2 hours on a plate shaker at RT then were washed twice in 0.05% PBS Tween 20. 40 μl of Streptavidin, R-Phycoerythrin Conjugate (SAPE) (#S866, Thermo Fisher) at 4 μg/ml was then added for 1 hour, followed by the addition of 10 μl of 10 μg/ml of R-Phycoerythrin, Biotin-XX Conjugate (#P811, Thermo Fisher), with incubation for an additional hour. Plates were then washed three times, sheath buffer was added to each well,

with plates left to shake for 10 minutes on a plate shaker prior to acquisition on a FlexMap3D™ (Luminex Corporation). The binding of ACE2, detected as phycoerythrin-labelled reporter is measured as MFI (Median Fluorescence Intensity).

Plaque reduction neutralization test (PRNT)

Plaque reduction neutralisation test (PRNT) was performed using clinical isolates of SARS-CoV-2 hCoV-19/Australia/VIC01/2020 and hCoV-19/Australia/VIC2089/2020. Duplicate two-fold serial dilutions of nanobody were prepared in Dulbecco's Modified Eagle (DME) media (ThermoFisher) and combined with an equal volume of DME media + 2 µg/ml trypsin tosyl phenylalanyl chloromethyl ketone (TPCK) (ThermoFisher) containing 180 TCID50 SARS-CoV-2. The nanobody/virus mixture was incubated at room temperature for one hour before plating onto confluent monolayers of Vero cells (clone CCL81) in 24-well plates and a further incubation at 37 °C supplied with 5% CO_2 for 1 hour. 0.6 mL of DME media containing 4% fetal bovine serum (FBS) + 1.5% (w/v) methylcellulose (Sigma) was added to each well and plates were incubated for five days at 37°C supplied with 5% CO_2. Plaques were visualised and counted by staining with 0.2% (w/v) crystal violet after fixation in 4% formaldehyde. IC50 values were calculated using four-parameter logistic regression using GraphPad Prism 8.0 (GraphPad Software Inc).

Measurement of viral burden for *in vivo* studies

Three days post-infection, animals were humanely killed and lungs removed and homogenised in a Bullet Blender (Next Advance Inc) in 1 mL DME media (ThermoFisher) containing steel homogenisation beads (Next Advance Inc). Samples were clarified by centrifugation at 10,000 *x* g for 5 minutes before virus quantification by TCID50 and RT-qPCR assays. SARS-CoV-2 live virus quantification by TCID50 assay: SARS-CoV-2 lung TCID50 was determined by plating 1:7 serially-diluted lung tissue homogenate onto confluent layers of Vero cells (clone CCL81) in DME media (ThermoFisher) containing 0.5 µg/ml trypsin-TPCK (ThermoFisher) in replicates of six on 96-well plates. Plates were incubated at 37 °C supplied with 5% CO_2 for four days before measuring cytopathic effect under light microscope. The TCID50 calculation was performed using the Spearman and Kärber method. SARS-CoV2 N1 gene copy number quantification by RT-qPCR: SARS-CoV-2 RNA was extracted from clarified lung homogenate using NucleoSpin 96-well plate virus extraction kits (Macherey-Nagel GmbH&Co). Primers and probes specific for the

conserved SARS-CoV2 N1 gene were purchased from Integrated DNA Technologies (Iowa, USA). Primer/probe sequences: 2019-nCoV_N1 forward primer – 5'-GAC CCC AAA ATC AGC GAA AT-3', 2019-nCoV_N1 reverse primer – 5'-TCT GGT TAC TGC CAG TTG AAT CTG-3', 2019-nCoV_N1 probe – 5'-FAM-ACC CCG CAT TAC GTT TGG TGG ACC-BHQ1-3'. Viral RNA was reverse transcribed and amplified using iTaq Universal Probe Kit (Bio-Rad Inc) and run on a LightCycler 96 machine (Roche). Cycle parameters: RT reaction – 600 seconds/50 °C, pol activation – 120 seconds/95 °C, denaturation – 15 seconds/95 °C, annealing – 60 seconds/60 °C. To determine absolute SARS-CoV-2 N1 gene copy number, standard curves were generated using a commercially validated standard control sample (SKU: COV019, Exact Diagnostics LLC). RT-qPCR data were analysed using LightCycler 480 software (Roche). To compare TCID50 and RT-qPCR values between groups we performed a Kruskal-Wallis test across the groups followed with Dunn's post test to obtain p-values comparing treated groups with controls or high dose animals.

Mass spectrometry sample preparation and analyses

Tryptic digestion of gel separated Nanobody proteins.

Nanobody bands were excised and processed. Briefly gel bands were first destained in a solution of 100 mM NH_4HCO_3 / 50% ethanol for 15 minutes at room temperature with shaking at 750 rpm. Destaining was repeated twice to ensure removal of excess Coomassie. Destained bands were dehydrated with 100% ethanol for 5 minutes and then rehydrated in 50 mM NH_4HCO_3 containing 10 mM DTT. Protein bands were reduced for 60 minutes at 56 °C with shaking then washed twice in 100% ethanol for 10 minutes to remove DTT. Reduced ethanol washed samples were sequentially alkylated with 55 mM iodoacetamide in 50 mM NH_4HCO_3 in the dark for 45 minutes at room temperature. Alkylated samples were then washed with milli-Q water followed by 100% ethanol twice for 5 minutes to remove residual Iodoacetamide then vacuum-dried for 10 minutes. Alkylated samples were then rehydrated with 20 ng/µl trypsin (Promega) in 50 mM NH_4HCO_3 at 4 °C for 1 hour. Excess trypsin was removed, gel pieces were covered in 40 mM NH_4HCO_3 and incubated overnight at 37 °C. Peptides were concentrated and desalted using C18 stage tips before analysis by LC-MS.

Identification of Nanobody derived peptides using reversed phase LC-MS.

Purified peptides were re-suspended in 0.1% TFA, 2% acetonitrile and separated using a two-column chromatography set up composed of a PepMap100 C18 20 mm x 75 µm trap and a PepMap C18 500 mm x 75 µm analytical column (Thermo Fisher Scientific). Samples were concentrated onto the trap column at 5 µL/min for 5 minutes with Buffer A (0.1% formic acid, 2% DMSO) and then infused into a Orbitrap Fusion™ Lumos™ Tribrid™ Mass Spectrometer (Thermo Fisher Scientific) equipped with a FAIMS Pro interface at 300 nl/minute via the analytical column using a Dionex Ultimate 3000 UPLC (Thermo Fisher Scientific). 125-minute gradients were run for each sample altering the buffer composition from 3% buffer B (0.1% formic acid, 77.9% acetonitrile, 2% DMSO) to 28% B over 95 minutes, then from 28% B to 40% B over 10 minutes, then from 40% B to 80% B over 7 minutes, the composition was held at 80% B for 3 minutes, and then dropped to 3% B over 0.1 minutes and held at 3% B for another 9 minutes. The Lumos™ Mass Spectrometer was operated in a stepped FAIMS data-dependent mode automatically switching between the acquisition of a single Orbitrap MS scan (120,000 resolution) every 2 seconds and HCD MS2 events (FTMS, 30K resolution, maximum fill time 80 ms, normalize collision energy 30, AGC of 250%) at three different FAIMS CVs -25, -45 and -65 as previously described (6). Carbohydrate associated oxonium ions (204.0867; 138.0545 and 366.1396 m/z) within HCD scans triggered product-dependent MS/MS analysis (7) with three additional scans to enhance the identification of potential glycopeptides; a EThcD (FTMS, 30K resolution, maximum fill time 350 ms, supplementary activation of normalize collision energy 25, AGC 500%) scan, a CID (ITMS, maximum fill time 35 ms, AGC 200%) scan and a stepped HCD (FTMS, 30K resolution, maximum fill time 250 ms, normalize collision energies of 28, 35 and 45 and a AGC of 250%).

The identification of nanobody associated peptides and glycopeptides was accomplished using Byonic (Protein Metrics, version 3.9.6) (8). The MS raw files were searched with a MS1 tolerance of ± 5 ppm and a tolerance of ± 20 ppm for HCD / EThcD MS2 scans. Searches were performed using cysteine carbamidomethylation as a fixed modification, methionine oxidation as a variable modification in addition to allowing *N*-linked glycosylation on asparagine residues. The default Byonic *N*-linked glycan database, which is composed of 309 mammalian *N*-glycans was used. The proteases specificity was

set to full trypsin specificity and a maximum of two miss-cleavage events. Data was searched against the expected nanobody protein sequences. Searches was filtered to a 1% protein FDR as set in the Byonic parameters. The mass spectrometry proteomics data have been deposited to the ProteomeXchange Consortium via the PRIDE partner repository (9, 10) with the dataset identifier PXD023483.

Flow cytometry-based syncytia formation assay

Expression of human ACE2 and SARS-CoV-2 spike protein in mouse embryonic fibroblasts

C57BL/6 mouse embryonic fibroblasts (MEFs) immortalized by transfection with DNA encoding SV40-large T-antigen, were passaged in Dulbecco's Modified Eagles Medium (DMEM) supplemented with 10% foetal calf serum (FCS), 55 µM 2-mercaptoethanol and 250 µM asparagine at 37 °C, 10% CO_2. Human ACE2 was cloned into the retroviral expression vector pMSCV-IRES-GFP and SARS-CoV-2 spike protein was cloned into retroviral pMSCV-IRES-mCherry, these were transduced into MEFs with packaging cell line HEK293T cells using standard protocols. SARS-CoV-2 spike and human ACE2 expressing MEF cells as well as MEFs transduced with retroviral particles derived from transfecting HEK293T with empty vector pMSCV-IRES-GFP (serving as negative controls for fusion) were sorted using flow cytometry (BD FACS fusion) based on expression levels of mCherry and GFP, respectively. Sorted cells stably expressing SARS-CoV-2 spike, human ACE2 and GFP were passaged in DMEM as outlined above.

Flow cytometry for syncytia formation

For syncytia formation inhibition assays, ten-fold WNbFc dilutions were prepared in duplicate by serially diluting each 80 mg/mL stock by 1:2 in DMEM. 1 x 10^4 MEFs expressing SARS-CoV-2 spike (mCherry) were seeded in 50 mL of DMEM into flat bottom 96-well tissue culture plates using a multichannel pipette, and 100 mL of the individual WNbFc dilutions were added to the MEFs expressing SARS-CoV-2 spike (mCherry) immediately after seeding. DMEM without added WNbFc served as maximum fusion control. After five minutes, 1 x 10^4 human ACE2 (GFP) in 50 mL DMEM were added to the wells to obtain a 1:1 ratio of ACE2 and spike expressing cells. MEFs expressing GFP only were used as background fusion control. The plates were kept for 24 hours at 37 °C, 5% CO_2. Next day, the wells were washed once with PBS, incubated with

trypsin for five minutes and the cells harvested in DMEM. The single cell suspensions were transferred into new U bottom 96-well plates. Fusion events (5000 events/well) were recorded using flow cytometry on a BD Fortessa1 or Fortessa x20 using automated HTS 96 well plate reader. Acquired data was analyzed using FlowJo 10.7, and syncytia were identified by gating on GFP+ mCherry+ cells. The percentage of maximum fusion and syncytia formation inhibition were calculated by subtracting the background value obtained from the wells with MEFs expressing SARS-CoV-2 spike (mCherry) and GFP control from all wells with MEFs expressing SARS-CoV-2 spike (mCherry) and ACE2 (GFP) without or with WNbFc dilutions, respectively.

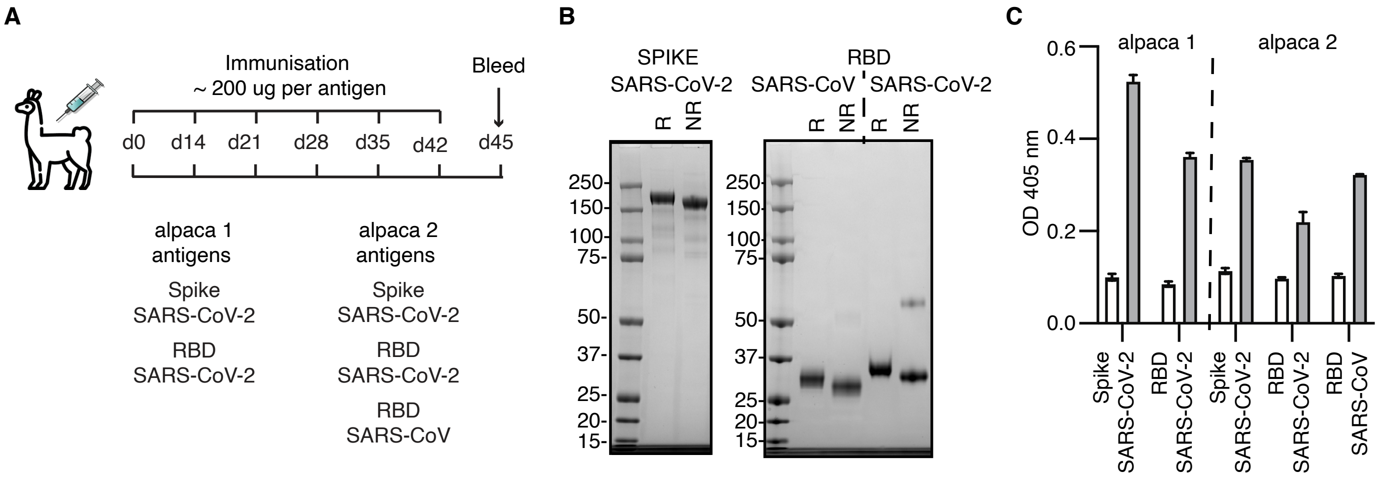

Fig. S1. Immunization of alpacas for generation of anti-SARS-CoV-2 nanobodies. (A) Two alpacas were immunised six times with the indicated antigens within a duration of 42 days. The alpacas were bled on d45 post-immunisation for post-immune sera and lymphocytes. (B) Recombinant antigens used for the immunisation ran on a SDS-PAGE under non-reducing (NR) and reducing (R) conditions. Molecular weight in kDa is shown on the left of both Coomasie-stained gels. (C) ELISA of the pre- and post-immunisation alpaca sera shows increased reactivity to the recombinant antigen after immunization. Reactivity to pre-immune sera and post-immune sera are shown in white and grey columns respectively. 125 nM of recombinant antigen was coated on the ELISA plates with 1:1000 dilution of polyclonal alpaca serum used. Error bars represent the mean ± SD of technical duplicates. The graph is a representative of two independent experiments.

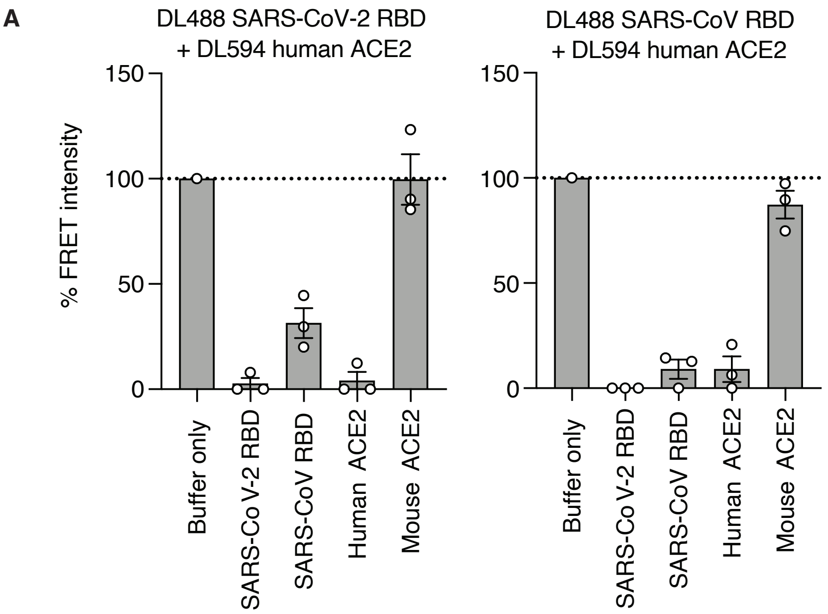

Fig. S2. FRET-based assay of human ACE2 - RBD interaction. Measured FRET fluorescence intensity of the interaction between SARS-CoV-2-Dylight488 (left panel) or SARS-CoV-Dylight488 (right panel) with human ACE2-Dylight594 incubated at 1:1 molar concentration either in buffer only or in the presence of unlabeled proteins (SARS-CoV-2 RBD, SARS-CoV RBD, human ACE2 and mouse ACE2). The fluorescence intensity (FI) of DyLight-488 (donor) was measured with a 485/14-nm excitation filter and a 535/25-nm emission filter and DyLight-594 was measured with a 590/20-nm excitation filter and 615/9-nm emission filter. The FRET signal was relative to "buffer only" control.

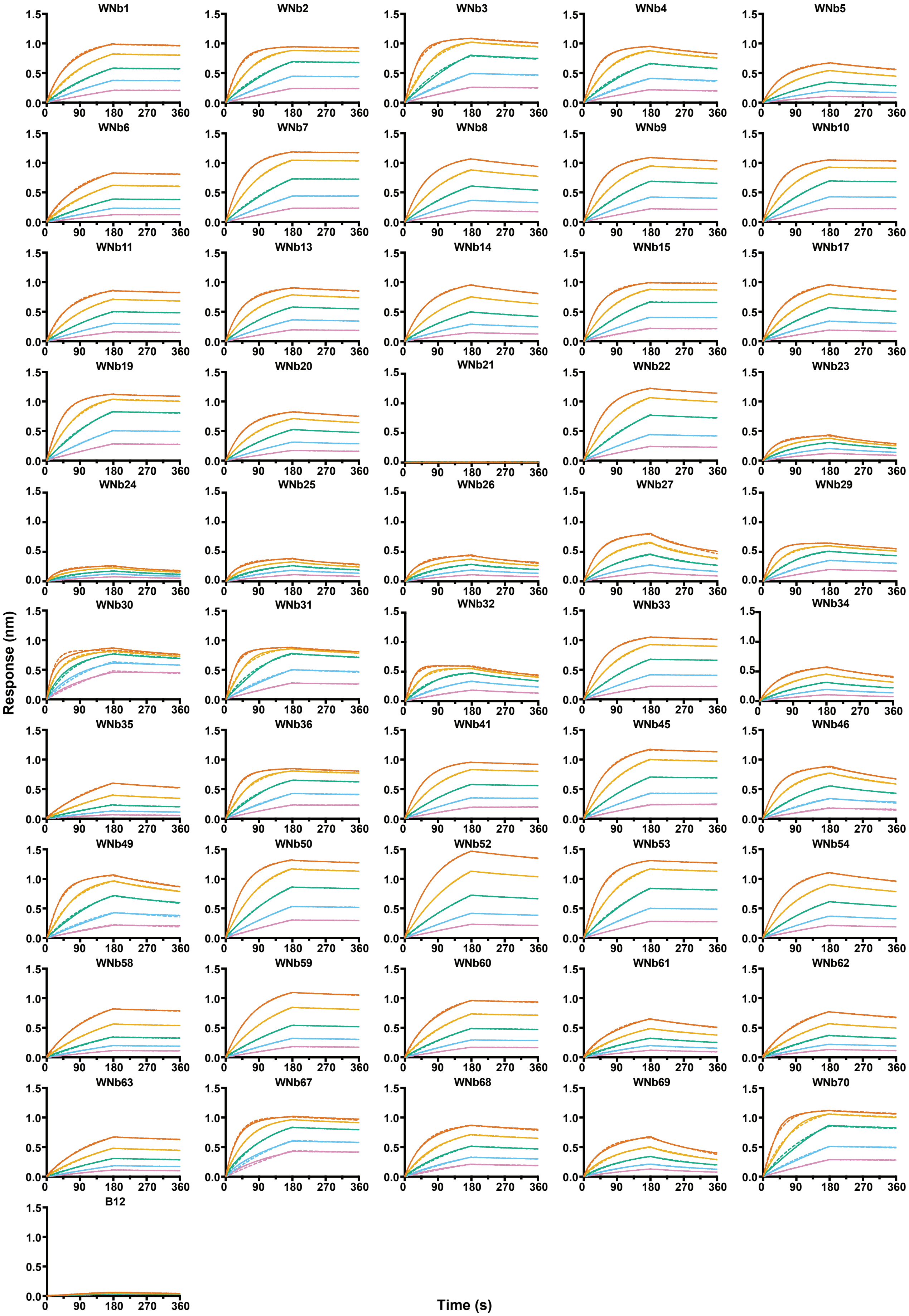

Fig. S3. Nanobody binding kinetics using bio-layer interferometry (BLI). BLI experiment with immobilized WNb nanobodies and SARS-CoV-2 RBD in solution. Binding curves of five different RBD concentration from 6 to 100 nM are plotted (solid line) and fitted to a 1:1 binding model (dashed line). Representative binding curves are shown from three independent experiments. B12 nanobody is specific to a *P. falciparum* antigen and represent a non-specific nanobody control

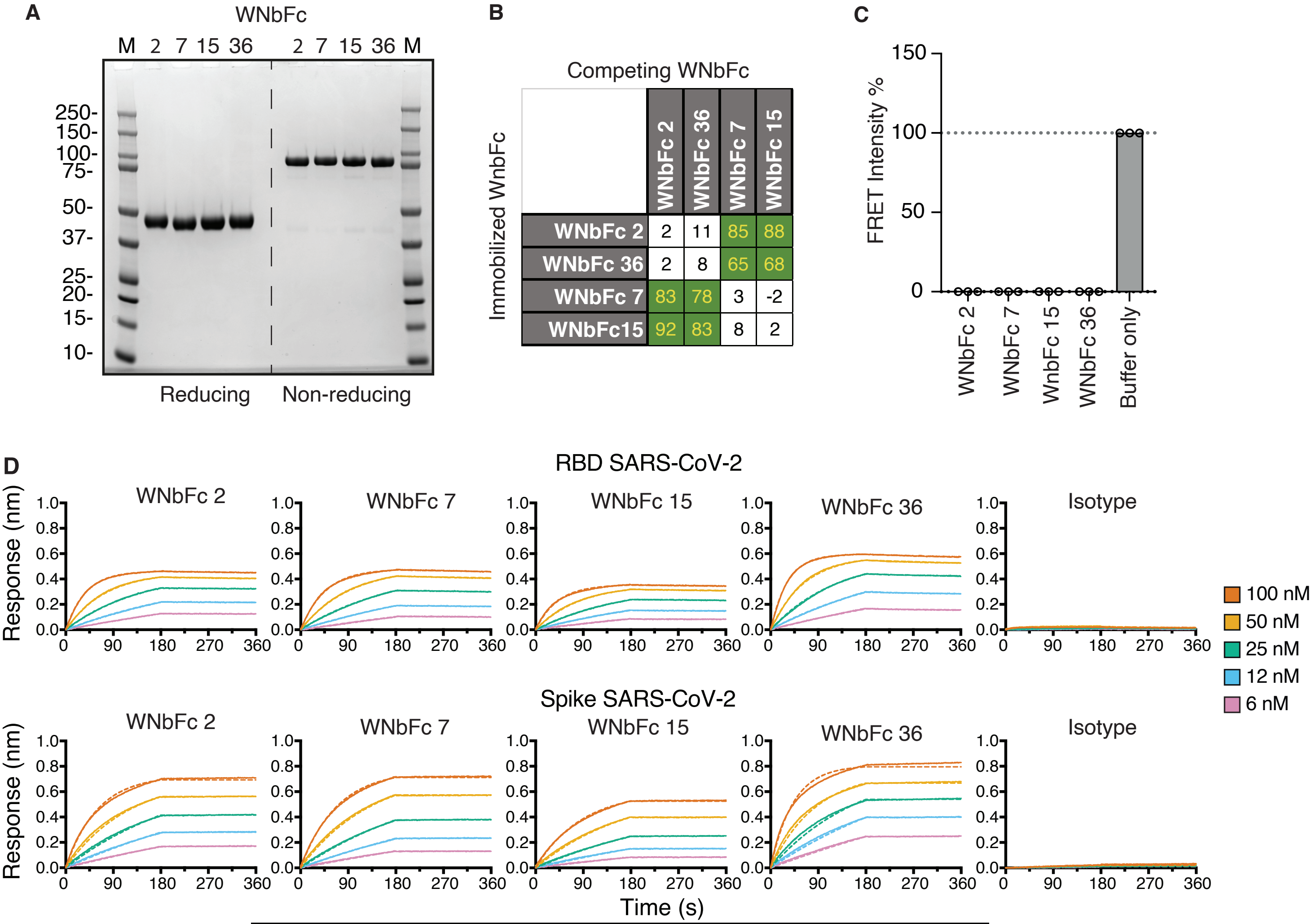

RBD SARS-CoV-2			
**Ab**	**K_D (nM)**	**k_a (x 10^5 $M^{-1}s^{-1}$)**	**k_d (x 10^{-5} s^{-1})**
WNb2-Fc	0.59 (± 0.07)	2.65 (± 0.27)	15.83 (± 3.23)
WNb7-Fc	0.70 (± 0.12)	2.76 (± 0.30)	19.10 (± 4.06)
WNb15-Fc	0.44 (± 0.15)	3.03 (± 0.31)	12.84 (± 4.35)
WNb36-Fc	0.61 (± 0.13)	3.39 (± 0.15)	20.20 (± 4.05)
Spike SARS-CoV-2			
**Ab**	**K_D (nM)**	**k_a (x 10^5 $M^{-1}s^{-1}$)**	**k_d (x 10^{-5} s^{-1})**
WNb2-Fc	< 0.01	3.77 (± 0.28)	< 0.1
WNb7-Fc	< 0.01	2.14 (± 0.47)	< 0.1
WNb15-Fc	< 0.01	1.78 (± 0.34)	< 0.1
WNb36-Fc	< 0.01	4.85 (± 0.11)	< 0.1

Fig. S4. Purification and characterization of WNbFc fusions. (A) Purified WNbFc fusions were separated by SDS-PAGE under reducing (R) and non-reducing (NR) conditions. Molecular weight marker (M) in kDa is shown on the left and right hand-side. (B) Epitope competition experiments by BLI using immobilized RBD SARS-CoV-2 on sensors that were then dipped first into WNbFc antibodies indicated on the left column and secondly into WNbFc antibodies indicated on the top row. Binding of the second WNbFc to RBD SARS-CoV-2 in the presence of the first WNbFc was calculated relative to the second WNbFc binding to RBD SARS-CoV-2 alone, which was assigned 100%. WNbFc fusions with more than 60% binding are considered non-competing and highlighted as green squares. (C) Measured FRET fluorescence intensity of the interaction between SARS-CoV-2-Dylight488 with human ACE2-Dylight594 incubated at 1:1 molar concentration either in buffer only or in the presence of WNbFc fusions. The FRET signal was relative to "buffer only" control. (D) Representative binding curves of five different RBD or spike concentrations from 6 – 100 nM to immobilized nanobodies are plotted (solid line) and were fitted to a 1:1 binding model (dashed line). Corresponding mean ± SEM K_D values are indicated and representative binding curves are shown from three independent experiments. Table containing kinetic and affinity data from three independent experiments showing the mean and standard error of the mean (SEM) of WNbFc fusions bound to SARS-CoV-2 RBD and Spike proteins.

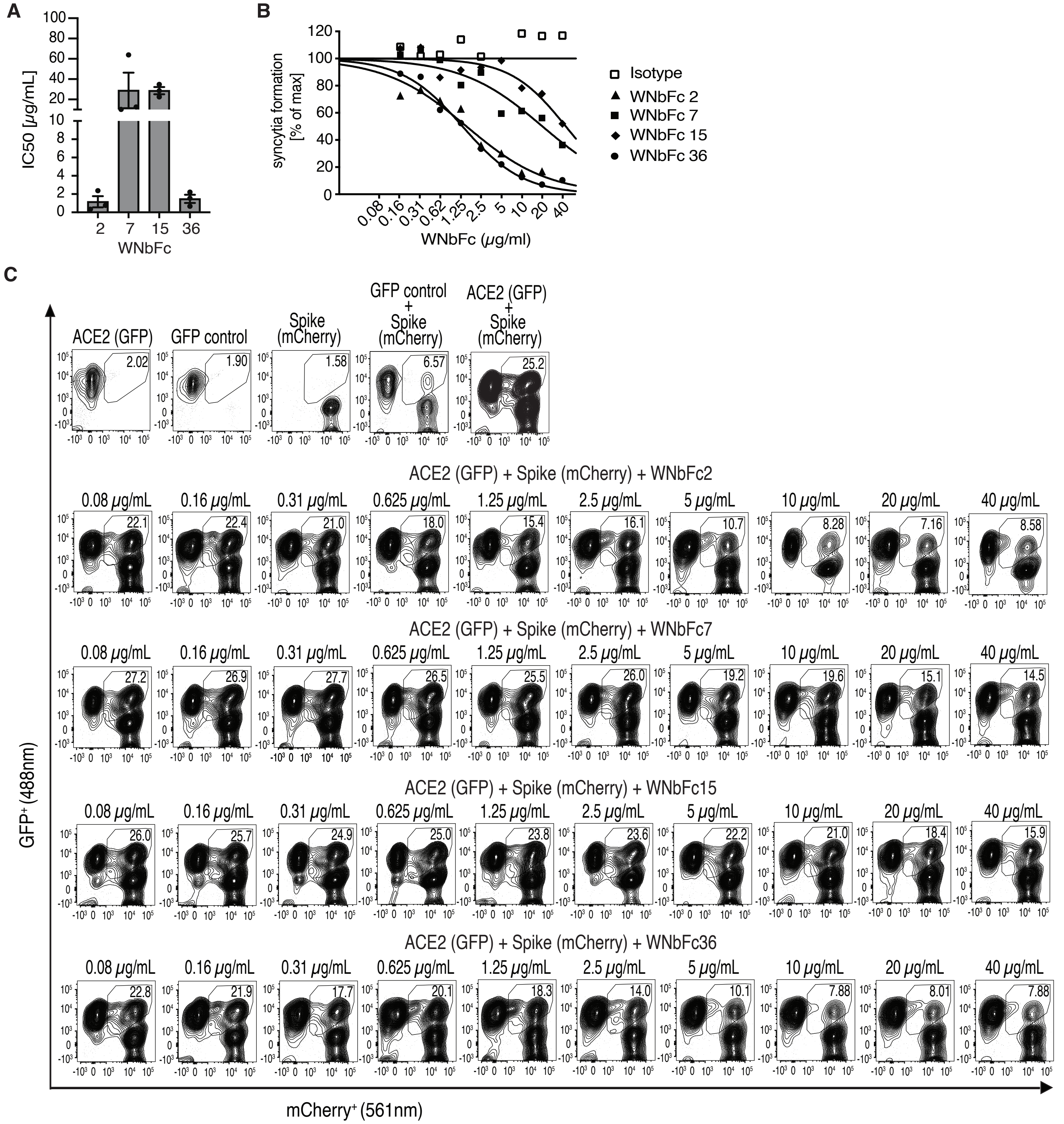

Fig. S5. Flow cytometry syncytia formation assay. (A) IC50 values for WNbFc inhibition of syncytia formation between MEFs expressing either human ACE2 (GFP) and SARS-CoV-2 Spike (mCherry). n=3 biological repeats done in duplicate per WNbFc concentration. Error bars represent SEM. **(B)** Representative IC50 curves for each WNbFc and isotype control based on dilution series ranging from 40 to 0.08 µg/mL. **(C)** Flow cytometry gating strategy showing representative flow plots of dilution series of all four WNbFc to determine the percentage of fused cells (GFP and mCherry double positive) and the subtracted background of non-specific fusion events using the GFP control and Spike (mCherry) condition.

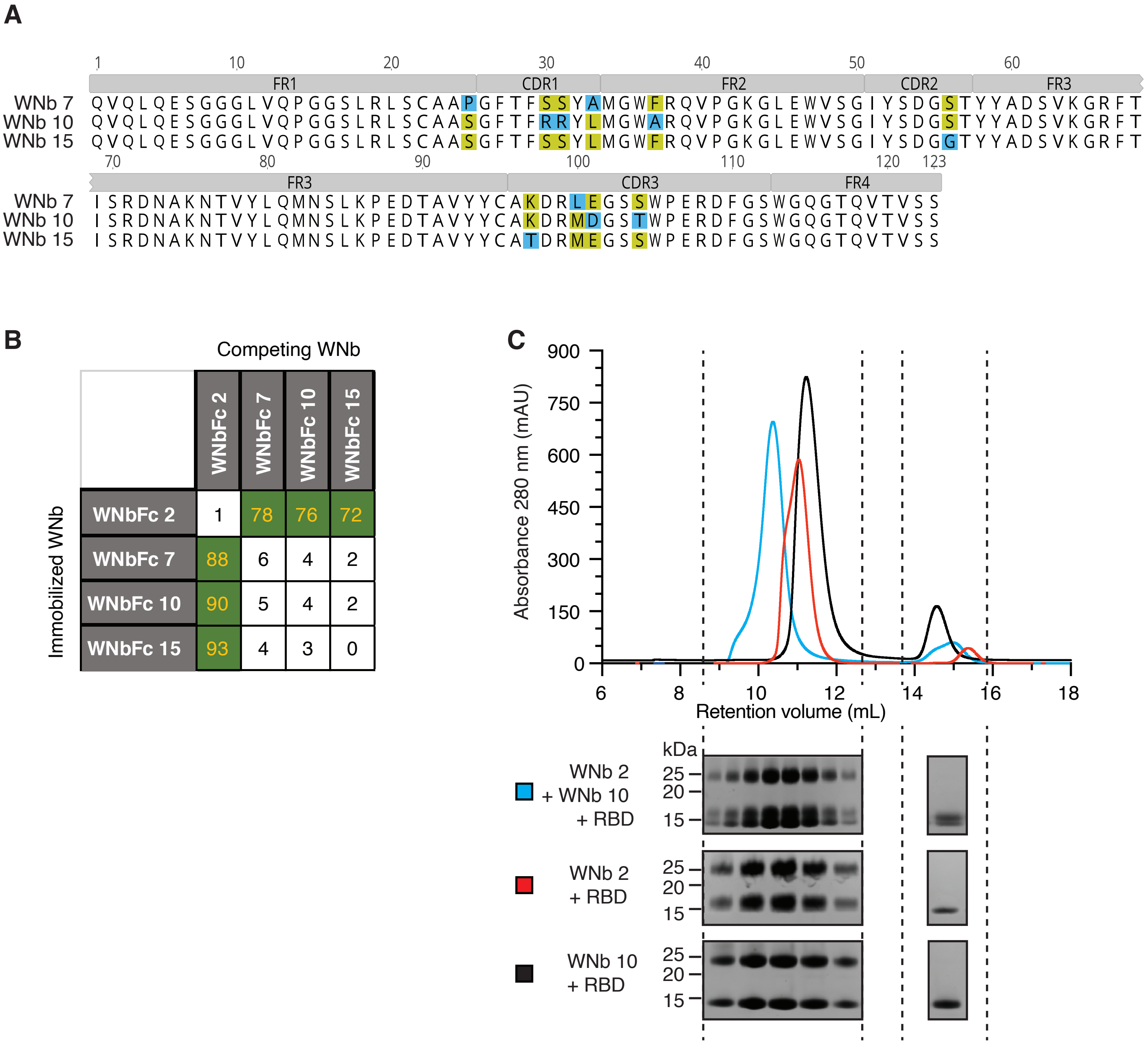

Fig. S6. WNb10 and WNb 2 bind to RBD simultaneously. (A) Alignment of WNb 7, 10 and 15 sequences with non-identical residues highlighted in green and blue. (B) Epitope competition experiments by BLI using immobilized RBD SARS-CoV-2 on sensors that were dipped first into WNbFc antibodies indicated on the left column and second into WNbFc antibodies indicated on the top row. Binding of the second WNbFc to RBD SARS-CoV-2 in the presence of the first WNbFc was calculated relative to the second WNbFc binding to RBD SARS-CoV-2 alone, which was assigned 100%. WNbFc fusions with more than 70% binding are considered non-competing and highlighted as green squares. (C) SEC analyses show that WNb 2 and WNb 10 binds to SARS-CoV-2 RBD simultaneously. The WNb 2 + WNb 10 + RBD complex elutes at a retention volume corresponding to higher molecular weight compared to the individual WNb 2 + RBD and the WNb 10 + RBD complexes on SEC. Excess of nanobodies elute as a second peak between retention volume of 14 to 16 mL.

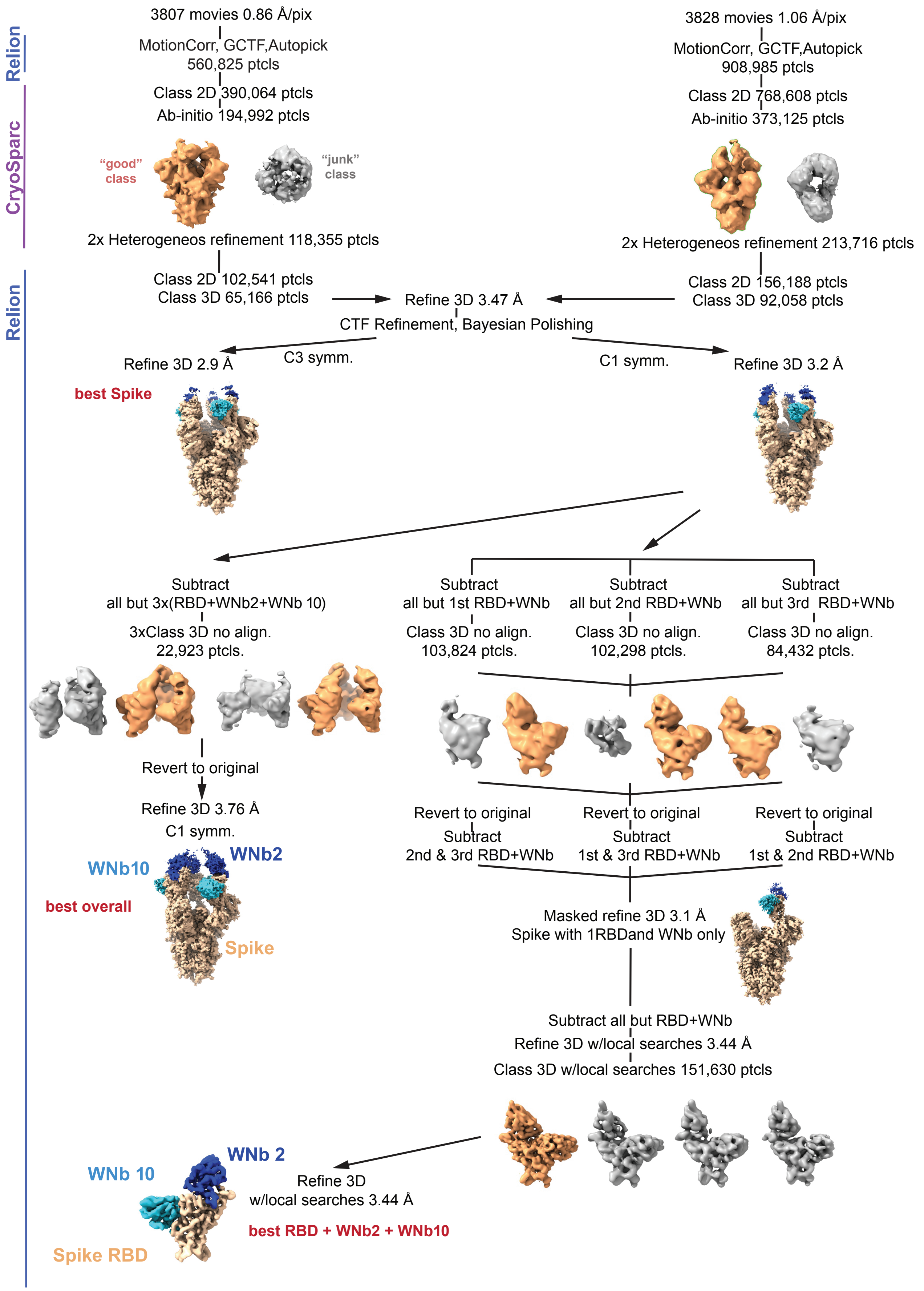

Fig. S7. Cryo-EM data processing workflow for Spike-WNb 2-WNb 10 complex. Details of workflow are fully described in materials and methods.

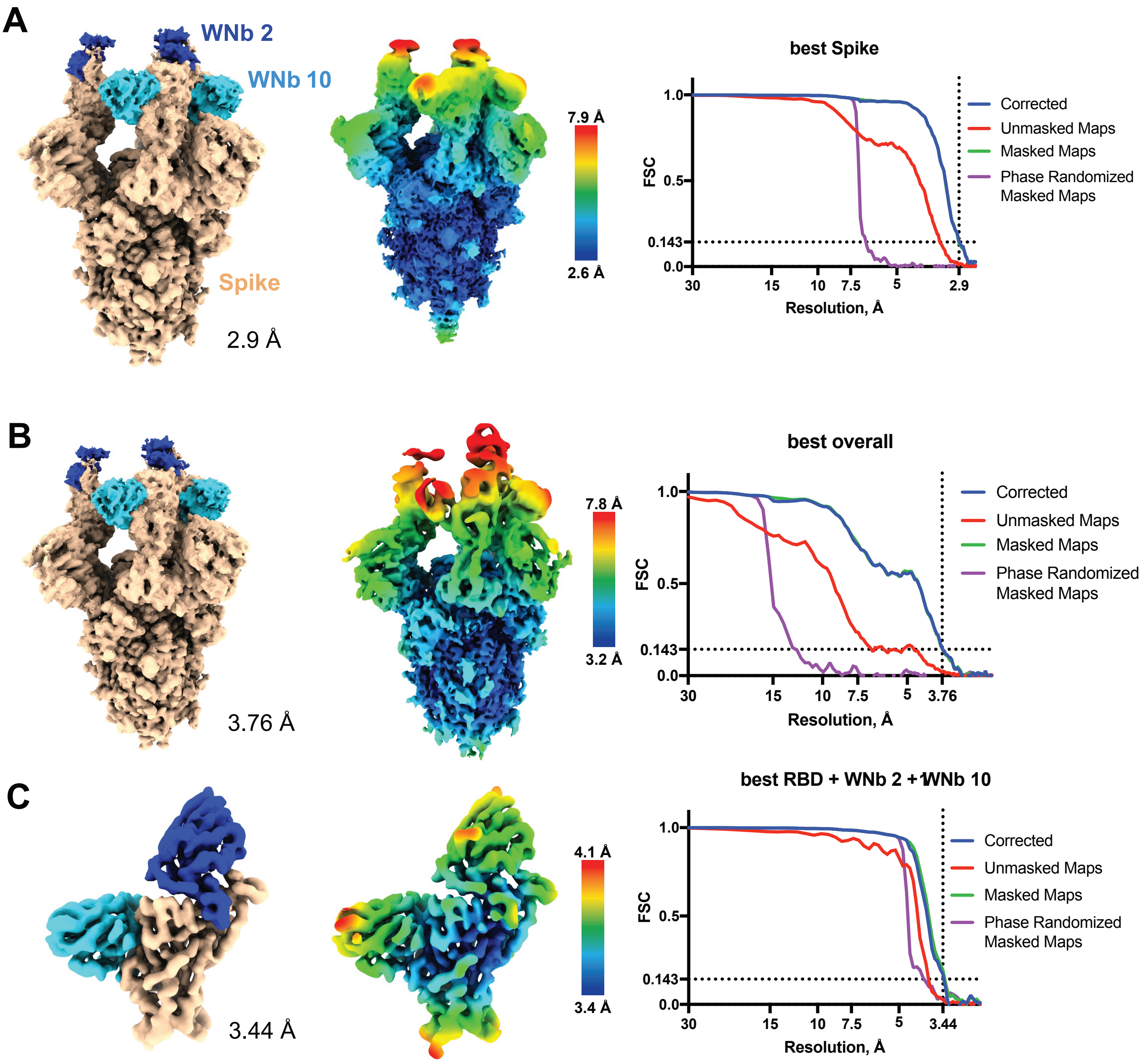

Fig. S8. Local resolution and FSC curves of cryo-EM maps. (A). "best Spike" map. **(B)** "best overall". **(C)** "best RBD + WNb 2+ WNb 10". Unsharpened maps from Refine 3D are on the left, local resolution maps are in the center and FSC curves are on the left.

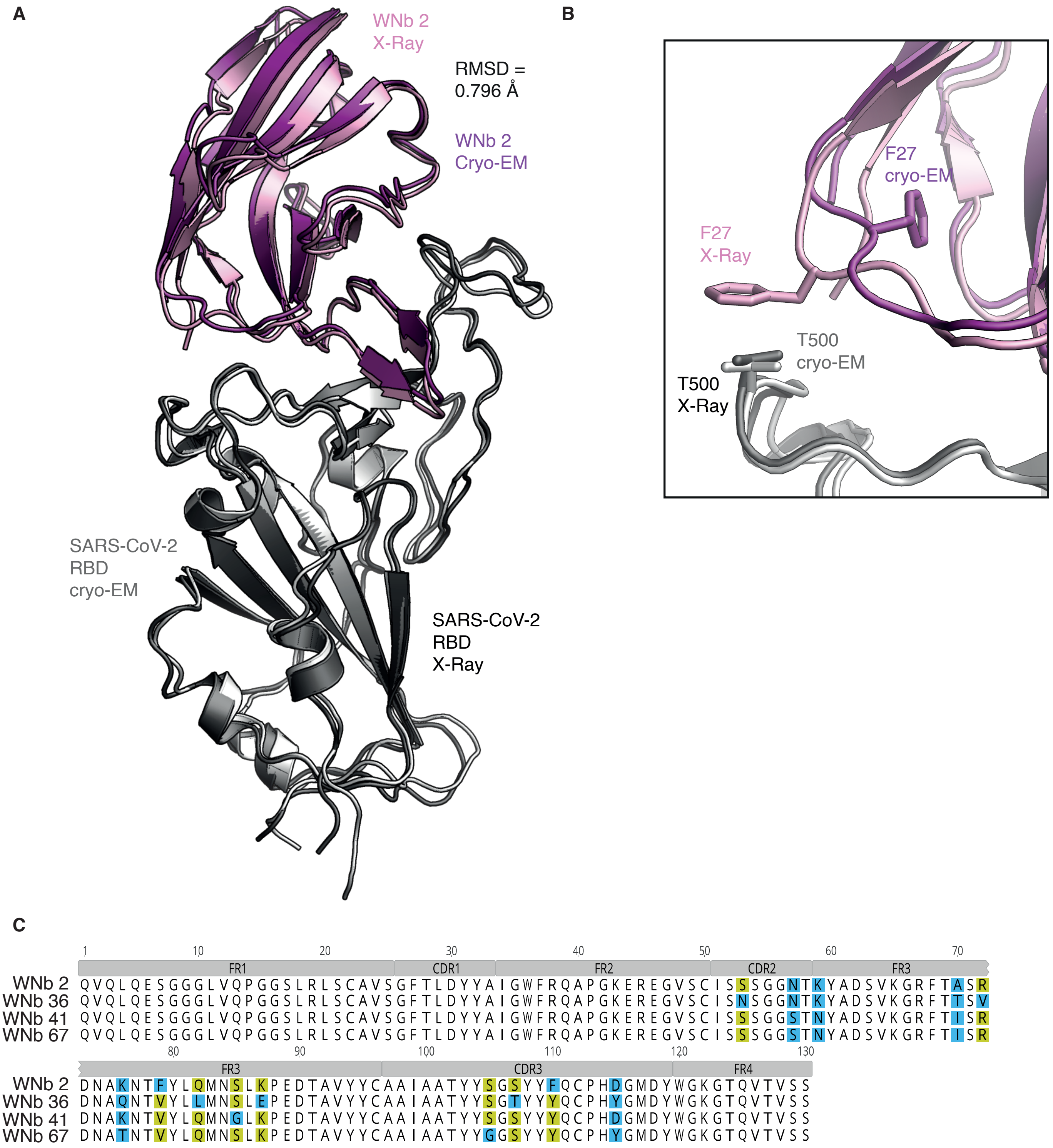

Figure S9: Overlay of the WNb 2 SARS-CoV-2 RBD cryo-EM structure with the WNb 2 SARS-CoV-2 RBD X-ray structure. (A) WNb 2 cryo-EM is coloured purple and WNb 2 X-ray structures is coloured pink, RBD cryo-EM is coloured charcoal and RBD X-ray structures is coloured silver. Overview of the complex aligned using least-squares-fit for SARS-CoV-2 RBD Cα's 331-527. **(B)** Close-up of WNb 2 residue F27 position in the cryo-EM and X-ray structures showing the interaction with T500 in the X-ray structure but not cryo-EM complex. (C) Alignment of WNb 2, 36, 41 and 67 sequences with non-identical residues highlighted in green and blue.

Table S1. WNb nanobody sequences

WNb ID	CDR1	CDR2	CDR3	Nanobody sequence
WNb 67	GFTLDYYA	ISSSGGST	AAIAATYYGGSYYYQCPHYGMDY	QVQLQESGGGLVQPGGSLRLSCAVSGFTLDYYAIGWFRQAPGKEREGVSCISSSGGSTNYADSVKGRFTISRDNATNTVYLQMNSLKPEDTAVYYCAAIAATYYGGSYYYQCPHYGMDYWGKGTQVTVSS
WNb 41	GFTLDYYA	ISSSGGST	AAIAATYYSGSYYYQCPHDGMDY	QVQLQESGGGLVQPGGSLRLSCAVSGFTLDYYAIGWFRQAPGKEREGVSCISSSGGSTNYADSVKGRFTISRDNAKNTVYLQMNGLKPEDTAVYYCAAIAATYYSGSYYYQCPHDGMDYWGKGTQVTVSS
WNb 36	GFTLDYYA	ISNSGGNT	AAIAATYYSGTYYYQCPHYGMDY	QVQLQESGGGLVQPGGSLRLSCAVSGFTLDYYAIGWFRQAPGKEREGVSCISNSGGNTKYADSVKGRFTTSVDNAQNTVYLLMNSLEPEDTAVYYCAAIAATYYSGTYYYQCPHYGMDYWGKGTQVTVSS
WNb 2	GFTLDYYA	ISSSGGNT	AAIAATYYSGSYYFQCPHDGMDY	QVQLQESGGGLVQPGGSLRLSCAVSGFTLDYYAIGWFRQAPGKEREGVSCISSSGGNTKYADSVKGRFTASRDNAKNTFYLQMNSLKPEDTAVYYCAAIAATYYSGSYYFQCPHDGMDYWGKGTQVTVSS
WNb 70	GFTLDFYA	ISSSGGSS	AAQMGSSEYCPIFIPGEGMDY	QVQLQESGGGLVQPGGSLNISCASNGFTLDFYAIGWFRQAPGKEREGVSCISSSGGSSKYSDSVKGRFTISRDNAKNTVYLQMNSLQPEDTAVYYCAAQMGSSEYCPIFIPGEGMDYWGKGTQVTVSS
WNb 4	GFTLDYYA	ISSSGGST	AAQMGSSRYCPIFTPGEGMDY	QVQLQESGGGLVQPGGSLRLSCAASGFTLDYYAIGWFRQAPGKEREGVSCISSSGGSTNYADSVKGRFTISRDNAKNTVYLQMNSLQPEDTAVYYCAAQMGSSRYCPIFTPGEGMDYWGKGTQVTVSS
WNb 3	GFTLDYYA	ISSSGGST	AAQMGSSRYCPIFIPGEGMDY	QVQLQESGGGLVQPGGSLRLSCAASGFTLDYYAIGWFRQAPGKEREGVSCISSSGGSTNYADSVKGRFTISRDNAKNTVYLQMNSLKPEDTAVYYCAAQMGSSRYCPIFIPGEGMDYWGKGTQVTVSS
WNb 59	GGTLDYYA	IMSSGGPT	AADPHGFDCSPSGGMDY	QVQLQESGGGLVQPGGSLRLSCAASGGTLDYYAIGWFRQAPGKEREGVSCIMSSGGPTYFAGSVKGRFTISRDNAKNTVYLQMNSLKPEDTAVYYCAADPHGFDCSPSGGMDYWGKGTQVTVSS
WNb 61	GFTLDFYA	ISRSGSNI	AATPSGVATAQGPCDLTRWYDN	QVQLQESGGGLVQPGGSLNISCASNGFTLDFYAIGWFRQAPGKEREGVSCISRSGSNINYAESLKGRATISRDNAKNTIYLQMSNLRTEDTAVYYCAATPSGVATAQGPCDLTRWYDNWGQGTQVTVSS
WNb 69	GFTLDYYG	ISASGTST	AAISGSYYYTSLHPVEYDS	QVQLQESGGGLVQPGGSLRLSCAASGFTLDYYGIGWFRQAPGKEREGVSCISASGTSTKYVNSVKGRFTISRDNAKNTVYLQMNSLKPEDTAVYYCAAISGSYYYTSLHPVEYDSWGQGTQVTVSS
WNb 46	GFTLDYYG	ISTSGTST	AAISGSYYYTSLHPVEYDY	QVQLQESGGGQVQPGGSLRLSCAASGFTLDYYGIGWFRQAPGQEREGVSSISTSGTSTNYANSVKGRFTISRDNAKNTVYLQMNSLKPEDTAVYYCAAISGSYYYTSLHPVEYDYWGQGTQVTVSS
WNb 49	GFTLDYYG	ISASGTST	AAISASYYYTSLHPVEYDS	QVQLQESGGGLVQPGGSLRLSCAASGFTLDYYGIGWFRQAPGKEREGVSGISASGTSTNYADSVKGRFTVSRDNAKNTVYLQMNSLKPEDTAVYYCAAISASYYYTSLHPVEYDSWGQGTQVTVSS
WNb 30	GITLDYYA	LSSSDGST	ATNPLTVVDGTFHLTCLDEYDY	QVQLQESGGGLVQPGGSLRLSCVASGITLDYYAIGWFRQAPGKEREGVSCLSSSDGSTYYADSVKGRFTMSRDNVKNTVYLQMNSLKPEDTAVYYCATNPLTVVDGTFHLTCLDEYDYGGQGTQVTVSS
WNb 20	GFTLEHYA	ISNSGGRT	AADLGVVERLCPAHPGSYEYDY	QVQLQESGGGLVQPGGSLRLSCAASGFTLEHYAIGWFRQAPGKEREGVSCISNSGGRTNYADSVKGRFTISRDNAKNTVYLQMNSLKPEDTAVYYCAADLGVVERLCPAHPGSYEYDYWGQGTQVTVSS
WNb 32	GRPLNSYS	IGRSDGLS	SAALWDGWATDINEYDY	QVQLQESGGGLVQAGGSLGLSCAASGRPLNSYSMAWFRQAPGKEREFVASIGRSDGLSKYNDFVKDRFTLSRGNAKTTLLLQMNNLKVEDTAVYYCSAALWDGWATDINEYDYRGQGTQVTVSS
WNb 31	GRALNTYT	IIWSSGAA	AVGQWEGRDTWYTSYDY	QVQLQESGGGLVQAGDSLRLSCTASGRALNTYTVAWWRQAPGNEREFVASIIWSSGAAWYADAVKGRFTISRDNAKNTVDLQMTSLKPEETAIYFCAVGQWEGRDTWYTSYDYWGQGTQVTVSS
WNb 21	ERTFGRYV	ISRSTGST	AADKDGGLEYDY	QVQLQESGGGLVQAGGSLTLTCAASERTFGRYVLAWFRQAPGKERELVARISRSTGSTFHADFVLGRFTISRDNAKNTVYLTMNNLKPEDTAVYYCAADKDGGLEYDYWGQGTQVTVSS
WNb 5	GRTFSRYA	ISPSGGST	AASTPSYHYCSGYVSEYDY	QVQLQESGGGLVQAGGSLRLSCAASGRTFSRYAIGWFRQAPGKEREGVSCISPSGGSTNYADSVKGRFTISRDNAKNTVYLQMNSLKPEDTAVYYCAASTPSYHYCSGYVSEYDYWGQGTQVTVSS
WNb 53	GFTFSSYL	IYSDGST	AKDRMEDSTWPERDFGS	QVQLQESGGGLVQPGGSLRLSCAASGFTFSSYLMGWARQVPGKGLEWVAGIYSDGSTYYADSVKGRFIISRDNAKNTVYLQMNSLKPEDTAVYYCAKDRMEDSTWPERDFGSWGQGTQVTVSS
WNb 33	GFTFSSYL	IYSDGST	AKDRMEGSTWPERDLGS	QVQLQESGGGLVQPGGSLRLSCAASGFTFSSYLMGWARQVPGKGLEWVAGIYSDGSTYYADSVKGRFIISRDNAKNTVYLQMNSLKPEDTAVYYCAKDRMEGSTWPERDLGSWGQGTQVTVSS
WNb 11	GGTFSSYL	IYSDGST	AKDRMEGSTWPERDFGS	QVQLQESGGGLVQAGDSLRLSCAASGGTFSSYLMGWARQVPGKGLEWVAGIYSDGSTYYADSVKGRFIISRDNAKNTVYLQMNSLKPEDTAVYYCAKDRMEGSTWPERDFGSWGQGTQVTVSS
WNb 68	GFTFSSYL	IYSDGST	AKDRMAGSRWPERDFGF	QVQLQESGGGLVQPGGSLRLSCAASGFTFSSYLMGWARQVPGKGLEWVAGIYSDGSTHYANSVKGRFTISRDNAKNTVYLQMNSLKPEDTAVYYCAKDRMAGSRWPERDFGFWGQGTQVTVSS
WNb 54	RFTFSSYA	IYSDGST	AKDRMEGSVWPERDFGS	QVQLQESGGGLVQPGGSLRLSCAASRFTFSSYAMGWARQVPGKGLEWVSGIYSDGSTYYADSVKGRFIISRDNAKNTVYLQMNSLKPEDTAVYYCAKDRMEGSVWPERDFGSWGQGTQVTVSS
WNb 14	GFTFSSYA	IYSDGST	AKDRMEGSSWPERDFGS	QVQLQESGGGLVQPGGSLRLSCAASGFTFSSYAMGWARQVPGKGLEWVSGIYSDGSTYYADSVKGRFIISRDNAKNTVYLQMNSLKPEDTAVYYCAKDRMEGSSWPERDFGSWGQGTQVTVSS
WNb 22	GFTFSSYA	IYSDGST	AKDRMDGSVWPERDFGS	QVQLQESGGGLVQPGGSLRLSCAASGFTFSSYAMGWARQVPGKGLEWLSGIYSDGSTYYADSVKGRFTISRDNAKNTVYLQMNSLKPEDTAVYYCAKDRMDGSVWPERDFGSWGQGTQVTVSS
WNb 9	GFTFSSYA	IYSDGST	AKDRMAGSSWPERDFGS	QVQLQESGGGLVQPGGSLRLSCAASGFTFSSYAMGWARQVPGKGLEWVSGIYSDGSTYYGDSVKGRFTISRDNAKNTVYLQMNSLKPEDTAVYYCAKDRMAGSSWPERDFGSWGQGTQVTVSS
WNb 7	GFTFSSYA	IYSDGST	AKDRLEGSSWPERDFGS	QVQLQESGGGLVQPGGSLRLSCAAPGFTFSSYAMGWFRQVPGKGLEWVSGIYSDGSTYYADSVKGRFTISRDNAKNTVYLQMNSLKPEDTAVYYCAKDRLEGSSWPERDFGSWGQGTQVTVSS
WNb 6	GFTFSSYA	IYSDGST	AKDRLEGSSRPERDFGS	QVQLQESGGGLVQPGGSLRLSCAASGFTFSSYAMGWFRQVPGKGLEWVSGIYSDGSTYYADSVKGRFTISRDNAKNTLYLQMNSLKPEDTAVYYCAKDRLEGSSRPERDFGSWGQGTQVTVSS
WNb 8	GFTFSSYA	IYSDGST	AKDRLESSSWPERDFGS	QVQLQESGGGLVQPGGSLRLSCAASGFTFSSYAMGWARQVPGKGLEWVSGIYSDGSTYYADSVKGRFTISRDNAKNTVYLQMNSLKPEDTAVYYCAKDRLESSSWPERDFGSWGQGTQVTVSS
WNb 52	GFIFSSYA	IYSDGST	AKDRLEGSTWPERDFGS	QVQLQESGGGLVQPGGSLRLSCAASGFIFSSYAMGWARQVPGKGLEWVSGIYSDGSTYYADSVKGRFTISRDNAKNTVYLQMNSLKPKDTAVYYCAKDRLEGSTWPERDFGSWGQGTQVTVSS
WNb 35	GFTFSSYA	IYSDGTT	AADRMEGSTWPERDFGS	QVQLQESGGGLVQPGGSLRLSCAASGFTFSSYAMGWARQVPGKGLEWVSGIYSDGTTYYADSVKGRFTISRDNAKNTVYLQMNSLKPEDTAVYYCAADRMEGSTWPERDFGSWGQGTQVTVSS
WNb 50	GFTFSSYA	IYSDGST	AADRMAGSRWPERDFGS	QVQLQESGGGLVQPGGSLRLSCAASGFTFSSYAMGWFRQVPGKGLEWVSGIYSDGSTYYADSVKGRFTISRDNAKNTVYLQMNSLKPEDTAVYYCAADRMAGSRWPERDFGSWGQGTQVTVSS
WNb 13	GFTFSSYA	IYSDGST	ATDRMAGSRWPERDFGS	QVQLQESGGGLVQPGGSLRLSCAASGFTFSSYAMGWFRQVPGKGLEWVSGIYSDGSTYYADSVKGRFTISRDNAKNTVYLQMNSLKPEDTAVYYCATDRMAGSRWPERDFGSWGQGTQVTVSS
WNb 58	GFTFSSYA	IYSDGST	AADRMDGSRWPERDFGS	QVQLQESGGGLVQPGGSLRLSCAASGFTFSSYAMGWFRQVPGKGLEWVSGIYSDGSTYYADSVKGRFTISRDNAKNTVYLQMNSLKPEDTAVYYCAADRMDGSRWPERDFGSWGQGTQVTVSS
WNb 45	GFTFSSYA	IYSDGST	AADRMEGSSWPERDFGS	QVQLQESGGGLVQPGGSLRLSCAASGFTFSSYAMGWFRQVPGKGLEWVSGIYSDGSTYYADSVKGRFTISRDNAKNTVYLQMNSLKPEDTAVYYCAADRMEGSSWPERDFGSWGQGTQVTVSS
WNb 60	GFTFSSYL	IYSDGST	ATDRMDGSSWPERDFGS	QVQLQESGGGLVQPGGSLRLSCAASGFTFSSYLMGWFRQVPGKGLEWVAGIYSDGSTYYADSVKGRFTISRDNAKNTVYLQMNSLKPEDTAVYYCATDRMDGSSWPERDFGSWGQGTQVTVSS
WNb 15	GFTFSSYL	IYSDGGT	ATDRMEGSSWPERDFGS	QVQLQESGGGLVQPGGSLRLSCAASGFTFSSYLMGWFRQVPGKGLEWVSGIYSDGGTYYADSVKGRFTISRDNAKNTVYLQMNSLKPEDTAVYYCATDRMEGSSWPERDFGSWGQGTQVTVSS
WNb 63	GFTFRRYL	IYSDGST	AADRMDGSTWPERDFGS	QVQLQESGGGLVQPGGSLRLSCAASGFTFRRYLMGWARQVPGKGLEWVSGIYSDGSTYYADSVKGRFTISRDNAKNTVYLQMNSLKPEDTAVYYCAADRMDGSTWPERDFGSWGQGTQVTVSS
WNb 10	GFTFRRYL	IYSDGST	AKDRMDGSTWPERDFGS	QVQLQESGGGLVQPGGSLRLSCAASGFTFRRYLMGWARQVPGKGLEWVSGIYSDGSTYYADSVKGRFTISRDNAKNTVYLQMNSLKPEDTAVYYCAKDRMDGSTWPERDFGSWGQGTQVTVSS
WNb 1	GRTFDRYA	IYSDGYT	AADRMDGSSWPERDFGS	QVQLQESGGGLVQAGGSLRLSCAASGRTFDRYAMGWFRQVPGKGLEWVSGIYSDGYTYYADSVKGRFTISRDNAKNTVYLQMNSLKPEDTAVYYCAADRMDGSSWPERDFGSWGQGTQVTVSS
WNb 23	GFTFKDYA	IYSDGNT	ATERHPQGAADFGS	QVQLQESGGGLVQPGGSLRLSCAASGFTFKDYAMAWARQVPGKGLEWVSGIYSDGNTYYADSVKGRFTISRDNAKNTVYLRMNSLKPEDTAVYYCATERHPQGAADFGSWGQGTQVTVSS
WNb 62	RRTFSTCV	IYSDGST	NLDGRYENLEDDY	QVQLQESGGGLVQAGGSLRLSCTASRRTFSTCVMGWARQVPGKGLEWVSGIYSDGSTYYADSVKGRFTISRDNAKNTVYLQMNSLKPEDTAVYYCNLDGRYENLEDDYWGQGTQVTVSS
WNb 19	GSMFSINV	ITRDGAT	NTNVDSVWGSRIDY	QVQLQESGGGLVQPGGSLRLSCAASGSMFSINVMGWYRQASGKQRELVAGITRDGATNYSDSVKGRFTISGDNAKNTVYLQMNSLKPEDTAVYYCNTNVDSVWGSRIDYFGQGTQVTVSS
WNb 17	GSIFSINV	ITSVGST	NAVSTFTQAMGGSPEHY	QVQLQESGGGLVQPGGSLRLSCAASGSIFSINVMGWYRQAPGKQRELVAAITSVGSTNYADSVKGRFTISRDNAENTVYLQMNSLKPEDTAVYYCNAVSTFTQAMGGSPEHYWGQGTQVTVSS
WNb 34	GRTFSRYA	VSWNGGNT	AAGGEPVVVIKLRNANEYDF	QVQLQESGGGLVEAGDSLRLSCAASGRTFSRYAMGWFRQAPGKEREFVAAVSWNGGNTYYADSVKGRFTISRDNAKNTLYLQMNSLKPEDTAVYYCAAGGEPVVVIKLRNANEYDFLGQGTQVTVSS
WNb 24	GRTFSRYA	VSWNGGNT	AAGGEPVVVIKLRNADEYDF	QVQLQESGGGLVQAGESLRLSCAASGRTFSRYAMGWFRQAPGKEREFVAAVSWNGGNTYYADSVKGRFTISRDNAKNTLYLQMNSLKPEDTAVYYCAAGGEPVVVIKLRNADEYDFLGQGTQVTVSS
WNb 27	GRTFSRYA	VSWNGGNT	VADRGESYYYNRDSEYEW	QVQLQESGGGLVEAGDSLRLSCAASGRTFSRYAMGWFRQAPGKEREFVAAVSWNGGNTYYADSVKGRFTISRDNAKNTLYLQMNSLKPEDTARYYCVADRGESYYYNRDSEYEWWGQGTQVTVSS
WNb 26	GRTFSPYA	MSWSSGK	GAGGEGYSRGRVNLQPDF	QVQLQESGGGLVQAGGSLRLSCVVSGRTFSPYAMGWFRQAPGKEREFVAGMSWSSGKYYADSVKGRFTISRDGAKNAVYLQMNSLKPEDTAVYYCGAGGEGYSRGRVNLQPDFWGQGTQVTVSS
WNb 25	GLTFSRAA	IHWRGLNI	AAIPFYYFDHISPFSRAAAY	QVQLQESGGGLVQAGGSLRLSCVTSGLTFSRAAMGWFRQAPEKEREFVASIHWRGLNIAYADSVKGRFTISRDDAKNTLYLQMNSLKTEDTAVYYCAAIPFYYFDHISPFSRAAAYWGQGTQVTVSS
WNb 29	GRTFSMYA	IDDGGGRT	AADMGFGYTIWADSYDY	QVQLQESGGGLVQPGGSLRLSCAFSGRTFSMYAMGWFRQAPGKEREFVAVIDDGGGRTYYADSVKGRFTISRDNAKNTVYLQMNSLKTEDTAVYCCAADMGFGYTIWADSYDYWGQGTQVTVSS

Table S2. Nanobody affinities, binding inhibition and neutralization potencies.

	Binding[a]		Binding Affinities[b]			Binding Inhibition[c]	Neutralization
	ELISA (OD 405 nm)		SARS-CoV-2 RBD (mean ± SEM)			SARS-CoV-2 RBD + human ACE2	WT SARS-CoV-2
WNb ID	SARS-CoV-2 RBD	SARS-CoV RBD	K_D (nM)	k_a (x 10^5 $M^{-1}s^{-1}$)	k_d (x 10^{-5} s^{-1})	FRET (relative to positive)	MNV (nM)
WNb 67	1.90	0.09	0.61 (± 0.16)	3.52 (± 0.12)	21.23 (± 4.73)	0.01	31.8
WNb 41	1.82	0.08	0.55 (± 0.27)	1.89 (± 0.07)	10.40 (± 5.28)	0.02	49.4
WNb 36	1.81	0.08	0.43 (± 0.21)	2.98 (± 0.14)	12.72 (± 6.41)	0.00	3.3
WNb 2	1.81	0.09	0.36 (± 0.05)	2.12 (± 0.52)	7.34 (± 2.00)	0.00	23.5
WNb 70	1.82	0.10	0.81 (± 0.13)	2.53 (± 0.05)	20.43 (± 3.33)	0.01	155.5
WNb 4	1.72	0.09	4.44 (± 1.54)	2.04 (± 0.50)	75.03 (± 2.47)	0.10	213.1
WNb 3	1.77	0.12	2.08 (± 0.68)	2.17 (± 0.54)	37.90 (± 1.19)	0.06	95.0
WNb 59	1.83	1.79	1.21 (± 0.34)	1.32 (± 0.03)	15.87 (± 4.49)	0.05	355.8
WNb 61	1.29	1.43	12.74 (± 1.07)	1.05 (± 0.05)	133.30 (± 6.12)	0.05	1432.2
WNb 69	1.70	0.10	19.49 (± 1.56)	1.66 (± 0.06)	321.70 (± 13.68)	0.06	541.6
WNb 46	1.70	0.09	6.20 (± 0.14)	2.25 (± 0.12)	139.30 (± 6.23)	0.00	153.1
WNb 49	1.73	0.10	4.31 (± 0.05)	2.40 (± 0.12)	103.60 (± 5.56)	0.00	159.1
WNb 30	1.87	0.44	1.07 (± 0.03)	5.23 (± 0.34)	55.43 (± 2.11)	0.00	167.0
WNb 20	1.75	1.68	2.59 (± 0.18)	1.74 (± 0.08)	45.23 (± 5.06)	0.28	166.9
WNb 32	1.66	1.79	3.92 (± 0.26)	4.83 (± 0.38)	187.00 (± 2.08)	0.29	136.4
WNb 31	1.70	0.72	1.25 (± 0.09)	3.59 (± 0.07)	44.80 (± 3.76)	0.22	94.9
WNb 21	0.09	1.60	N/A	N/A	N/A	1.13	36108.9
WNb 5	1.79	0.63	8.52 (± 2.80)	1.36 (± 0.33)	97.83 (± 4.62)	0.00	215.0
WNb 53	1.75	1.80	0.64 (± 0.15)	2.01 (± 0.04)	12.98 (± 3.06)	0.00	57.4
WNb 33	1.77	1.81	0.73 (± 0.15)	1.92 (± 0.03)	13.94 (± 2.76)	0.03	161.2
WNb 11	1.68	1.83	1.96 (± 0.39)	1.40 (± 0.33)	25.57 (± 5.78)	0.03	97.5
WNb 68	1.82	1.82	1.75 (± 0.56)	1.87 (± 0.03)	32.80 (± 10.38)	0.02	219.5
WNb 54	1.57	1.68	4.57 (± 0.34)	1.66 (± 0.03)	75.37 (± 4.15)	0.00	217.0
WNb 14	1.42	1.47	8.89 (± 3.37)	1.28 (± 0.31)	93.33 (± 6.51)	0.02	389.7
WNb 22	1.64	1.61	1.88 (± 0.11)	1.78 (± 0.06)	33.57 (± 2.54)	0.02	86.8
WNb 9	1.66	1.70	2.35 (± 0.99)	1.64 (± 0.40)	30.73 (± 3.03)	0.00	98.0
WNb 7	1.73	1.86	0.26 (± 0.14)	1.67 (± 0.40)	3.19 (± 0.80)	0.02	54.5
WNb 6	1.71	1.61	1.21 (± 0.19)	0.99 (± 0.19)	11.19 (± 1.32)	0.03	109.1
WNb 8	1.48	1.60	6.62 (± 2.60)	1.37 (± 0.33)	73.30 (± 5.40)	0.08	218.4
WNb 52	1.68	1.72	3.57 (± 0.26)	1.24 (± 0.03)	44.17 (± 2.67)	0.00	175.5
WNb 35	1.24	1.44	12.64 (± 0.98)	0.60 (± 0.01)	75.60 (± 5.51)	0.07	1322.6
WNb 50	1.82	1.86	0.63 (± 0.15)	2.06 (± 0.04)	12.99 (± 3.35)	0.00	60.1
WNb 13	1.66	1.76	2.12 (± 0.67)	1.96 (± 0.55)	34.67 (± 3.04)	0.01	97.1
WNb 58	1.73	1.82	1.91 (± 0.63)	0.87 (± 0.03)	16.72 (± 5.66)	0.03	559.6
WNb 45	1.72	1.80	0.68 (± 0.09)	1.72 (± 0.03)	11.77 (± 1.57)	0.01	158.3
WNb 60	1.77	1.84	0.51 (± 0.45)	1.26 (± 0.05)	6.21 (± 5.51)	0.00	149.3
WNb 15	1.81	1.86	0.14 (± 0.06)	1.87 (± 0.44)	3.05 (± 1.59)	0.00	43.6
WNb 63	1.79	1.84	2.83 (± 1.05)	0.86 (± 0.06)	23.57 (± 7.97)	0.00	332.6
WNb 10	1.75	1.82	0.68 (± 0.27)	1.75 (± 0.43)	9.64 (± 0.70)	0.02	68.1
WNb 1	1.76	1.85	0.79 (± 0.10)	1.48 (± 0.30)	11.13 (± 1.54)	0.00	96.4
WNb 23	0.58	1.90	9.03 (± 0.21)	2.72 (± 0.08)	245.30 (± 10.71)	0.22	1812.5
WNb 62	1.50	1.47	6.41 (± 0.93)	1.03 (± 0.05)	65.17 (± 7.49)	0.02	1205.8
WNb 19	1.73	1.77	0.46 (± 0.14)	2.28 (± 0.10)	10.66 (± 3.56)	0.00	71.2
WNb 17	1.50	1.62	3.77 (± 0.21)	1.58 (± 0.04)	59.77 (± 4.75)	0.03	280.2
WNb 34	1.79	1.49	11.05 (± 0.73)	1.73 (± 0.07)	190.00 (± 5.13)	0.61	1709.8
WNb 24	1.66	0.28	7.33 (± 0.22)	3.02 (± 0.10)	220.70 (± 0.33)	0.91	749.9
WNb 27	1.65	0.17	12.33 (± 0.61)	2.36 (± 0.10)	289.70 (± 2.60)	0.73	370.5
WNb 26	1.70	0.65	7.38 (± 0.22)	2.67 (± 0.10)	197.00 (± 2.52)	0.65	869.4
WNb 25	1.69	0.35	6.02 (± 0.28)	2.89 (± 0.13)	173.00 (± 0.58)	0.69	370.9
WNb 29	1.69	1.27	2.33 (± 0.08)	3.87 (± 0.15)	89.77 (± 1.44)	0.28	170.7

[a]Average values from n=2 biological replicates all with two technical replicates

[b]Values from n=3 bio-layer interferometry experiments

[c]Average values from n=3 biological replicates all with triplicate technical replicates. The FRET signal was relative to "positive" control, which has no added nanobody, which was normalized to the value of 1.

Table S3. Cryo-EM data collection, refinement and validation statistics

	Best Spike +WNb2+WNb10 (EMDB EMD-23567)	Best overall +WNb2+WNb10 (EMDB EMD-23568)	RBD +WNb2 +WNb10 (localized reconstruction) (EMDB EMD-23566) (PDB 7LX5)
**Data collection and processing**			
Magnification	165K	165K	165K
Voltage (kV)	300 keV	300 keV	300 keV
Electron exposure (e–/Å^2)	1	1	1
Defocus range (μm)			
Pixel size (Å)	0.86 and 1.06	0.86 and 1.06	0.86 and 1.06
Symmetry imposed	C3	C1	C1
Initial particle images (no.)	1,489,810	1,489,810	1,489,810
Final particle images (no.)	152,750	22,923	151,630
Map resolution (Å)	2.9	3.76	3.44
FSC threshold	0.143	0.143	0.143
Map resolution range (Å)	2.6-7.9	3.2-7.8	3.4-4.1
**Refinement**	-	-	7LDJ
Initial model used (PDB code)			
Model resolution (Å)			3.66
FSC threshold			0.5
Map sharpening *B* factor (Å^2)			-60
Model composition			
Non-hydrogen atoms			3480
Protein residues			448
Ligands			-
B factors (Å^2)			
Protein (min-max (avr))			63.5-149 (120.8)
Ligand			-
R.m.s. deviations			
Bond lengths (Å)			0.005
Bond angles (°)			0.604
Validation			
MolProbity score			1.44
Clashscore			4.73
Poor rotamers (%)			0
Ramachandran plot			
Favored (%)			96.83
Allowed (%)			3.17
Disallowed (%)			0

Table S4 | Data collection and refinement statistics for SARS-CoV-2 RBD WNb 2 Complex

	SARS-CoV-2 RBD WNb 2 (PDB 7LDJ)
**Data collection**	
Space group	P 1 2_1 1
Cell dimensions	
a, *b*, *c* (Å)	73.12, 88.51, 107.49
α, β, γ (°)	90.00, 90.40, 90.00
Resolution (Å)	44.25 - 2.36 (2.44 - 2.36)*
R_{merge}	0.030 (0.382)
$I/\sigma(I)$	13.00 (1.84)
$CC_{1/2}$	0.999 (0.803)
Completeness (%)	99.9 (100.0)
Redundancy	7.0 (7.3)
**Refinement**	
Resolution (Å)	44.25 - 2.36
No. reflections	56465
R_{work} / R_{free} (%)	21.66/ 27.30
No. atoms	
Protein	10060
Ligand/ion	205
Water	164
B factors	
Protein	58.89
Ligand/ion	81.82
Water	54.22
R.m.s. deviations	
Bond lengths (Å)	0.004
Bond angles (°)	0.690
Validation	
MolProbity score	1.57
Clashscore	5.57
Poor rotamers (%)	0.56
Ramachandran plot	
Favored (%)	96.1
Allowed (%)	3.63
Disallowed (%)	0.32

X-ray diffraction data were collected on single crystals.
* Values in parentheses are for highest-resolution shell.

Table S5 | Summary of interactions between SARS-CoV-2 RBD and nanobodies WNb 2 and WNb 10.

SARS-CoV-2 RBD and WNb 2 nanobody based on the crystal structure PDB 7LDJ

SARS-CoV-2 RBD	Group	WNb 2	Group	Distance (Å)
		Hydrogen bonds		
Gly 446	O	Asp 30	N	3.0
Tyr 449	OH	Asp 30	N	3.4
Tyr 449	OH	Tyr 31	N	2.8
Tyr 449	OH	Tyr 31	O	2.7
Phe 490	O	Gln 111	NE2	3.1
Phe 490	N	Gln 111	OE1	2.6
Leu 492	O	Thr 102	OG1	3.3
Gln 493	NE2	Ala 100	O	2.8
Ser 494	N	Thr 102	OG1	3.1
Ser 494	OG	Tyr 103	N	3.1
Gln 498	NE2	Phe 27	O	3.3
Tyr 505	OH	Asp 118	O	3.2
		Salt bridges		
Arg 403	NH1	Asp 118	OD1	3.3
Arg 403	NH2	Asp 118	OD2	3.5
Lys 417	NZ	Asp 115	OD2	3.1
Glu 484	OE2	Lys 59	NZ	3.1

Other SARS-CoV-2 RBD interfacing residues (WNb 2)					
Gln 1	Thr 28	Leu 29	Gly 47	Val 48	Ser 49
Tyr 60	Ala 61	Ile 99	Ala 101	Tyr 104	Tyr 109
Pro 113	Gly 116	Tyr 119			

Other WNb 2 interfacing residues (SARS-CoV-2 RBD)					
Tyr 351	Leu 452	Tyr 453	Leu 455	Phe 456	Thr 470
Phe 486	Tyr 489	Tyr 495	Thr 500	Asn 501	

SARS-CoV-2 RBD and WNb 10 nanobody based on the Cryo-EM structure PDB XXX

SARS-CoV-2 RBD	Group	WNb 10	Group	Distance (Å)
		Hydrogen bonds		
Tyr 369	O	Arg 108	NH2	3.1
Ser 371	OH	Arg 108	NH2	3.6
Ala 372	OH	Trp 47	N	3.0
Phe 374	OH	Arg 108	NE	3.0
Thr 376	O	Asp 109	OD1	3.2
Phe 377	N	Arg 108	O	2.8
Phe 377	O	Arg 108	N	3.2
Tyr 508	OH	Trp 113	NE1	3.5

Other SARS-CoV-2 RBD interfacing residues (WNb 10)					
Gln 39	Lys 43	Leu 45	Glu 46	Tyr 58	Tyr 94
Arg 99	Met 100	Pro 106	Glu 107	Phe 110	

Other WNb 10 interfacing residues (SARS-CoV-2 RBD)					
Leu 368	Ser 375	Lys 378	Pro 384	Arg 408	Asn 437
Asn 440	Val 503	Gln 506			

* SARS-CoV-2 receptor binding domain residues that contact ACE2 (green).

SI References

1. E. Pardon, *et al.*, A general protocol for the generation of Nanobodies for structural biology. *Nat Protoc* **9**, 674–693 (2014).

2. F. Amanat, *et al.*, A serological assay to detect SARS-CoV-2 seroconversion in humans. *Nat Med* **26**, 1033–1036 (2020).

3. D. Wrapp, *et al.*, Cryo-EM structure of the 2019-nCoV spike in the prefusion conformation. *Science* **367**, 1260–1263 (2020).

4. J. A. Juno, *et al.*, Humoral and circulating follicular helper T cell responses in recovered patients with COVID-19. *Nat Med* **26**, 1428–1434 (2020).

5. K. J. Selva, *et al.*, Distinct systems serology features in children, elderly and COVID patients. *medRxiv*, 2020.05.11.20098459 (2020).

6. A. R. Ahmad Izaham, *et al.*, What Are We Missing by Using Hydrophilic Enrichment? Improving Bacterial Glycoproteome Coverage Using Total Proteome and FAIMS Analyses. *J Proteome Res* **20**, 599–612 (2021).

7. J. Saba, S. Dutta, E. Hemenway, R. Viner, Increasing the productivity of glycopeptides analysis by using higher-energy collision dissociation-accurate mass-product-dependent electron transfer dissociation. *Int J Proteomics* **2012**, 560391 (2012).

8. M. Bern, Y. J. Kil, C. Becker, Byonic: advanced peptide and protein identification software. *Curr Protoc Bioinformatics* **Chapter 13**, Unit13.20 (2012).

9. Y. Perez-Riverol, *et al.*, The PRIDE database and related tools and resources in 2019: improving support for quantification data. *Nucleic Acids Res* **47**, D442–D450 (2019).

10. J. A. Vizcaíno, *et al.*, 2016 update of the PRIDE database and its related tools. *Nucleic Acids Res* **44**, 11033 (2016).
